# Accelerometer-measured physical activity, frailty, and all-cause mortality and life expectancy among middle-aged and older adults: a UK Biobank longitudinal study

**DOI:** 10.1186/s12916-025-03960-z

**Published:** 2025-02-27

**Authors:** Yang Yang, Liangkai Chen, Filippos T. Filippidis

**Affiliations:** 1 Department of Primary Care and Public Health, School of Public Health, Imperial College London, London, W12 0BZ UK; 2 Department of Nutrition and Food Hygiene, Hubei Key Laboratory of Food Nutrition and Safety, School of Public Health, Tongji Medical College, Huazhong University of Science and Technology, Wuhan, China; 3 Ministry of Education Key Lab of Environment and Health, School of Public Health, Tongji Medical College, Huazhong University of Science and Technology, Wuhan, China

**Keywords:** Physical activity, Frailty, Mortality, Life expectancy, UK Biobank

## Abstract

**Background:**

Physical activity (PA) is associated with reduced frailty and lower mortality rates among middle-aged and older adults. However, the extent to which total PA volume and specific PA intensities are associated with mortality risk across frailty status remains unclear. We aimed to investigate the interactive effects of accelerometer-measured PA with frailty on all-cause mortality and life expectancy.

**Methods:**

A total of 78,508 participants were sourced from the UK Biobank for analysis. Frailty index (FI) was used to assess frailty status. Physical activity and sedentary behavior were quantified through accelerometer measurements, capturing the total volume of physical activity (TVPA), moderate-to-vigorous-intensity physical activity (MVPA), light-intensity physical activity (LPA), and sedentary time (ST). Cox proportional hazard models were applied to calculate adjusted hazard ratios (HRs) and predict life expectancy.

**Results:**

During a median follow-up of 6.9 years, 2618 deaths (2.9%) were identified. Compared with robust and physically active counterparts, individuals characterized by frailty, combined with the lowest levels of TVPA (HR 3.05, 95% CI: 2.50–3.71), MVPA (HR 2.65, 95% CI: 2.19–3.21), LPA (HR 2.26; 95% CI: 1.81–2.83), or the highest level of ST (HR 2.08, 95% CI: 1.66–2.61), were found to have the greatest risk of all-cause mortality after comprehensive adjustment. The dose–response relationship, assessed using restricted cubic splines, consistently demonstrated that regardless of frailty categories, higher levels of TVPA, MVPA, and LPA were associated with lower mortality risks, while higher ST level was associated with increased risk. Notably, across the frailty spectrum, individuals in the low tertile of TVPA, MVPA, and LPA, or the top tertile of ST, were associated with reduced life expectancy, with this pattern being more pronounced among frail men compared to frail women.

**Conclusions:**

Our findings highlighted the importance of increasing total PA volume, emphasizing MVPA and LPA, and reducing ST across the frailty spectrum to improve life expectancy.

**Supplementary Information:**

The online version contains supplementary material available at 10.1186/s12916-025-03960-z.

## Background

The global demographic landscape is undergoing a seismic shift towards an aging population, a trend projected to gain momentum in the coming decades. According to the World Health Organization, the global population of individuals aged 60 and above is anticipated to double by 2050, reaching an estimated 2 billion [1]. Aging of the population is leading to a marked increase in chronic conditions, subsequently elevating the prevalence of frailty [2]. It is estimated that the overall prevalence of pre-frailty and frailty in the UK is 37.5% and 3.3%, respectively, in middle-aged and older adults [3].

Frailty, a medical syndrome marked by age-associated declines in functioning across various physiological systems, increases vulnerability to stressors and poses an elevated risk for adverse health outcomes [4]. Frailty has been documented to be associated with a greater risk of falls, hospitalizations, disabilities, mortality, and a lower quality of life [5–7]. The escalating financial burden of long-term home care due to increased reliance on family caregiving for individuals losing daily living abilities, combined with the direct costs to social services and medical attention, underscores the urgent need for strategies to prevent frailty and its associated morbidities.

Physical activity (PA) has been highlighted as one of the most cost-effective strategies in the management of frailty and other chronic conditions [8]. Recent studies increasingly differentiate PA into distinct metrics, including total PA volume and specific activity intensities such as moderate-to-vigorous physical activity (MVPA), light physical activity (LPA), and sedentary time (ST) [9]. Yet, significant uncertainties persist regarding whether and to what extent, the known associations between PA and health outcomes apply to individuals with frailty. This uncertainty raises critical question about the feasibility and potential applicability of PA for those in advanced stages of frailty, potentially due to their physical limitations. Exploring how PA influences frail populations is vital, as it helps determine the optimal timing for interventions, and understand its association with all-cause mortality and life expectancy. Moreover, the potential heterogeneity in associations of intensity-specific PA (including MVPA, LPA, and ST) with mortality risk across frailty levels has not been fully explored. While total physical activity volume provides a comprehensive measure of overall movement, analyzing different intensity domains allows for a more nuanced understanding of physiological mechanisms and evaluates whether these intensities have distinct associations with mortality across varying frailty levels. Additionally, existing studies predominantly rely on self-reported measures of PA, which introduce methodological limitations such as recall and social desirability biases, often leading to overestimation of PA levels [10, 11]. These measures are typically restricted to specific PA domains (e.g., leisure-time activity), tend to underestimate ST [12], and rarely provide comprehensive data on total PA and LPA [11].

Consequently, this study aims to address these gaps by utilizing accelerometer-based measurements to examine the independent and interactive effects of PA/ST with frailty on all-cause mortality and life expectancy in middle-aged and older adults.

## Methods

### Study population

The UK Biobank is a large-scale, population-based cohort study in the UK. Between 2006 and 2010, the UK Biobank enrolled over 500,000 community-dwelling adults, aged 37–73, at 22 assessment centers throughout the UK. The study amassed comprehensive phenotypic and genotypic data for each participant. A detailed account of the UK Biobank’s methodology has been published elsewhere [13]. Ethical approval for the UK Biobank study was granted by the North West Multi-Center Research Ethics Committee (REC reference 21/NW/0157). All participants provided written informed consent prior to their involvement. The current research utilized the UK Biobank Resource under Application Numbers 63424 and 88,159.

From February 2013 to December 2015, the UK Biobank invited 236,519 participants to undergo PA assessments by wearing a wrist-worn Axivity AX3 accelerometer device (Axivity, Newcastle upon Tyne, UK). Accelerometer data were collected for seven consecutive 24-h periods. Of these invitees, 106,053 consented to participate, and 103,712 raw accelerometer datasets were received [14].

After initial processing, 11,528 participants were excluded due to low-quality data or insufficient wear time (less than 3 days of valid monitoring or no data on both Saturday and Sunday). We further excluded those lacking complete data for constructing a 49-item frailty index (*n* = 13,483) and for all-cause mortality (*n* = 5). Additionally, participants younger than 45 at the time of accelerometer measurement were excluded (*n* = 188). Ultimately, 78,508 participants were included in the main analysis. A detailed flowchart of the selection process is presented in Additional file 1: Fig. S1, and baseline characteristics of participants with and without complete frailty index data are compared in Additional file 1: Table S1.

### Accelerometer-measured physical activity

Participants used the Axivity AX3 wrist-worn triaxial accelerometer, a commercial version of the Open Movement AX3 sensor developed by Open Lab at Newcastle University (https://github.com/digitalinteraction/openmovement), for objective assessment of physical activity. Raw data processing was conducted by the UK Biobank accelerometer expert working group, following a protocol outlined in the prior publication [14, 15]. PA was quantified using the Euclidean Norm Minus One (ENMO) in milli-gravity (mg) to measure movement-related acceleration [16]. Subsequently, a validated machine learning model, employing balanced random forests with Hidden Markov models, was utilized to categorize movement behaviors into 30-s intervals, including moderate-to-vigorous-intensity physical activity (MVPA), light-intensity physical activity (LPA), and sedentary time (ST) [17]. The total volume of physical activity (TVPA) was summarized as the average ENMO while awake (defined as 06:00 to 22:00). Daily MVPA and LPA were calculated as the cumulative minutes spent in activities with the metabolic equivalent of tasks (METs) values of ≥ 3 and 1.5–2.9, respectively, during a typical day [17]. Correlations between accelerometer-measured PA and ST are shown in Additional file 1: Fig. S2. TVPA, MVPA, LPA, and ST were categorized into tertiles, with the first tertile as the reference group.

### Frailty

The Fried frailty phenotype and Rockwood frailty index are commonly used measurements in frailty research [18]. This study utilized the frailty index (FI) due to its more accurate mortality predictions, better reproducibility and responsiveness, greater strength in maintaining consistency and adaptability across varied items, and superior differentiation capabilities, particularly at the lower to middle end of the frailty continuum [18, 19]. Designing and validating a standard FI in UK Biobank has been reported elsewhere [20]. Briefly, deficits were required to meet specific criteria: they needed to be age-related, associated with poor health, more prevalent in older individuals, neither rare nor universal, cover multiple areas of human functioning, and be available for at least 80% of participants [20]. The full list of deficits in this study is provided in Additional file 1: Table S2. The FI comprised 49 variables obtained from touch-screen questionnaires and nurse-led interviews at baseline (self-reported), covering health conditions, illnesses, physical limitations, and mental well-being [20]. Categorical variables were dichotomized (no deficit = 0; deficit = 1), and ordinal variables were mapped onto a score between zero and one. The FI was calculated by dividing the sum of present deficits by the total number of possible deficits, yielding values between zero and one; higher values indicated increased frailty [20]. We classified participants into three levels based on the prior study: robust (FI ≤ 0.12), pre-frailty (FI 0.12–0.24), or frailty (FI ≥ 0.24).

### Covariates

All participants completed a touchscreen questionnaire and anthropometric assessment at recruitment into the main study. Some also participated in up to two additional touchscreen interviews. Consistent with previous research [21, 22], we used the accelerometry time-point as the analytical baseline. Exceptions to this were sex and Townsend Index of deprivation that were only obtained at recruitment, ethnicity (self-reported), and family medical history where a condition was counted at any of the measurement points [21, 22]. The values of other covariates, including education level, employment stats, household income, smoking status, alcohol consumption, healthy diet score, sleep duration, and body mass index (BMI) were obtained from touchscreen questionnaires at the time-point closest to the accelerometry [21, 22]. Detailed information on the source and definition of covariates, assessment timeline, and missing percentages are shown in Additional file 1: Table S3–S5 and Fig. S3.

### Ascertainment of mortality and life expectancy

The primary outcome of this study was all-cause mortality. Follow-up time was calculated as person-time in months for each participant from the complete date of accelerometer wear to death, the date of losing follow-up, or the end of the study. Mortality records were identified by linking to death registries of the National Health Service (NHS) Information Centre for participants from England and Wales and the NHS Central Register Scotland for participants from Scotland. At the time of our analyses, mortality data were available up to 30 September 2021 for England and Wales and 31 October 2021 for Scotland.

To calculate the life expectancy of participants we used life tables [23]. We built the lifetables starting at age 50 years and ending at age 100 years with the following three estimates to calculate the cumulative survival from 50 years onward: (i) sex- and age-specific population mortality rate from the Office for National Statistics [24]; (ii) sex-specific hazard ratios (HRs) of all-cause mortality in FI, accelerometer-measured PA/ST, and their interactive combinations; (iii) sex-specific prevalence of each frequency of FI, accelerometer-measured PA/ST, and their interaction.

We fitted multivariable-adjusted Cox regression models for each gender separately to calculate the age-specific hazard ratios for all-cause mortality. The estimated life expectancy (years) from different levels was calculated at any given age for the reference group and each of the exposure groups. Details of the methods used for estimating life expectancy have been described in Additional file 1: Supplementary Method.

### Statistical analysis

Descriptive characteristics by frailty status are presented as frequencies with percentages for categorical variables and mean with standard deviations (SD) for continuous variables. Missing data of covariates were imputed via the R package “missForest” [25].

Cox proportional hazard regression models were employed to assess the hazard ratios (HRs) and corresponding 95% confidence intervals (CIs), using follow-up time as the underlying timescale. The proportional hazards assumption was verified through statistical tests and graphical diagnostics based on scaled Schoenfeld residuals, revealing no violations. Models were constructed to adjust for potential confounders: age at accelerometer measurement, sex, assessment center, body mass index, ethnicity, education, employment, household income, Townsend deprivation index, smoking status, alcohol drinking frequency, sleep duration, healthy diet score, family history of diabetes, family history of cardiovascular disease (CVD), family history of cancer, seasonality and total wear days. MVPA and LPA models were further adjusted for ST, the ST model was further adjusted for MVPA, while the TVPA model was not further adjusted. We found no collinearity issues through a correlation matrix analysis and the calculation of the variance inflation factor.

Three types of analyses were performed using multivariate Cox models to examine the combined effects of accelerometer-measured PA/ST and FI on all-cause mortality. First, we analyzed both the additive and multiplicative interactions between the accelerometer-measured PA/ST and the FI with all-cause mortality. In stratified analyses, restricted cubic splines of changes in accelerometer-measured PA/ST stratified by FI categories were applied to graphically estimate the effects, with 4 knots placed at the 5th, 35th, 65th, 95th percentiles (reference was the 5th percentile). Last, the joint association was subsequently investigated by stratifying the overall sample into nine mutually exclusive groups based on levels of accelerometer-measured PA/ST and categories of FI. We evaluated interactions between accelerometer-measured PA/ST and FI using likelihood ratio tests comparing models with and without a cross-product term.

A series of sensitivity analyses were performed. First, to mitigate the risk of reverse causation, we excluded participants who died within the first 2 years of follow-up.

Second, recognizing that the current accelerometer processing algorithm, which defines waking time as 06:00–22:00, may not accurately capture physical activity patterns for shift workers, we performed an analysis excluding shift workers. Third, two additional analyses were conducted by redefining the awake times from 07:00 to 21:00 and from 08:00 to 20:00. Fourth, to isolate independent effects, we further mutually adjusted for MVPA and LPA. Fifth, to address the issue of missing data, we reran the analysis excluding those with missing data for any covariate.

All statistical analyses were conducted using R software version 4.3.1. We used Monte Carlo simulation (parametric bootstrapping) with 10,000 runs to calculate the CIs of the life expectancy estimation with the boot R package. All statistical tests were two-sided, and we considered a *P* value of less than 0.05 to be statistically significant.

## Results

### Baseline characteristics

The baseline characteristics of the included participants, stratified by FI, are presented in the imputed dataset (Table 1) and the original dataset (Additional file 1: Table S6). Of the 90,320 participants, 3193 (3.5%) were identified as frail. During a median follow-up of 6.9 years, 2618 participants (2.9%) died. Baseline characteristics stratified by TVPA, MVPA, LPA, and ST are detailed in Additional file 1: Table S7–S10.

**Table 1:** Baseline characteristics of the participants stratified by frailty index

	**Overall**	**Robust**	**Pre-frailty**	**Frailty**
	(*N* = 78,508)	(*N* = 49,736)	(*N* = 25,579)	(*N* = 3193)
Age at accelerometer measurement, mean (SD)	62.0 (7.8)	61.5 (7.8)	62.8 (7.7)	63.5 (7.4)
Sex, *n* (%)				
Women	43,538 (55.5%)	26,757 (53.8%)	14,844 (58.0%)	1937 (60.7%)
Men	34,970 (44.5%)	22,979 (46.2%)	10,735 (42.0%)	1256 (39.3%)
Body mass index (BMI), kg/m^2^, *n* (%)				
< 25.0	31,356 (39.9%)	22,181 (44.6%)	8574 (33.5%)	601 (18.8%)
25.0–29.9	32,136 (40.9%)	20,400 (41.0%)	10,604 (41.5%)	1132 (35.5%)
≥ 30.0	15,016 (19.1%)	7155 (14.4%)	6401 (25.0%)	1460 (45.7%)
Ethnicity, *n* (%)				
White	76,355 (97.3%)	48,403 (97.3%)	24,867 (97.2%)	3085 (96.6%)
Other	2153 (2.7%)	1333 (2.7%)	712 (2.8%)	108 (3.4%)
Education, *n* (%)				
College or university degree	35,182 (44.8%)	23,967 (48.2%)	10,272 (40.2%)	943 (29.5%)
Secondary school	28,803 (36.7%)	17,785 (35.8%)	9748 (38.1%)	1270 (39.8%)
Primary school	6203 (7.9%)	3065 (6.2%)	2605 (10.2%)	533 (16.7%)
Professional qualification	8320 (10.6%)	4919 (9.9%)	2954 (11.5%)	447 (14.0%)
Employment, *n* (%)				
Employed	42,369 (54.0%)	28,455 (57.2%)	12,736 (49.8%)	1178 (36.9%)
Retired	32,111 (40.9%)	19,354 (38.9%)	11,320 (44.3%)	1437 (45.0%)
Inactive	4028 (5.1%)	1927 (3.9%)	1523 (6.0%)	578 (18.1%)
Household income, £/year, *n* (%)				
Less than 18,000	12,472 (15.9%)	6105 (12.3%)	5199 (20.3%)	1168 (36.6%)
18,000 to 30,999	19,679 (25.1%)	11,733 (23.6%)	7035 (27.5%)	911 (28.5%)
31,000 to 51,999	21,841 (27.8%)	14,352 (28.9%)	6841 (26.7%)	648 (20.3%)
52,000 to 100,000	18,315 (23.3%)	12,858 (25.9%)	5070 (19.8%)	387 (12.1%)
Greater than 100,000	6201 (7.9%)	4688 (9.4%)	1434 (5.6%)	79 (2.5%)
Townsend deprivation index, mean (SD)	− 1.79 (2.78)	− 1.92 (2.70)	− 1.64 (2.85)	− 0.842 (3.21)
Smoking status, *n* (%)				
Never	45,193 (57.6%)	30,325 (61.0%)	13,416 (52.4%)	1452 (45.5%)
Previous	28,468 (36.3%)	16,758 (33.7%)	10,316 (40.3%)	1394 (43.7%)
Current	4847 (6.2%)	2653 (5.3%)	1847 (7.2%)	347 (10.9%)
Alcohol drinking frequency, *n* (%)				
≥ 3 times/week	38,041 (48.5%)	25,335 (50.9%)	11,667 (45.6%)	1039 (32.5%)
< 3 times/week	35,795 (45.6%)	21,963 (44.2%)	12,084 (47.2%)	1748 (54.7%)
Never	4672 (6.0%)	2438 (4.9%)	1828 (7.1%)	406 (12.7%)
Sleep duration, *n* (%)				
7–8 h/day	56,048 (71.4%)	37,687 (75.8%)	16,790 (65.6%)	1571 (49.2%)
< 7 h/day	17,197 (21.9%)	9241 (18.6%)	6755 (26.4%)	1201 (37.6%)
> 8 h/day	5263 (6.7%)	2808 (5.6%)	2034 (8.0%)	421 (13.2%)
Healthy diet score, *n* (%)				
0–2	27,185 (34.6%)	16,652 (33.5%)	9294 (36.3%)	1239 (38.8%)
3–5	47,762 (60.8%)	30,724 (61.8%)	15,213 (59.5%)	1825 (57.2%)
≥ 6	3561 (4.5%)	2360 (4.7%)	1072 (4.2%)	129 (4.0%)
Family history of diabetes, *n* (%)				
No	60,987 (77.7%)	39,430 (79.3%)	19,338 (75.6%)	2219 (69.5%)
Yes	17,521 (22.3%)	10,306 (20.7%)	6241 (24.4%)	974 (30.5%)
Family history of CVD, *n* (%)				
No	17,621 (22.4%)	12,218 (24.6%)	4956 (19.4%)	447 (14.0%)
Yes	60,887 (77.6%)	37,518 (75.4%)	20,623 (80.6%)	2746 (86.0%)
Family history of cancer, *n* (%)				
No	49,063 (62.5%)	31,475 (63.3%)	15,646 (61.2%)	1942 (60.8%)
Yes	29,445 (37.5%)	18,261 (36.7%)	9933 (38.8%)	1251 (39.2%)
Total wear days, mean (SD)	6.72 (0.709)	6.73 (0.706)	6.72 (0.718)	6.73 (0.691)
Seasonality, *n* (%)				
Autumn	23,285 (29.7%)	14,854 (29.9%)	7472 (29.2%)	959 (30.0%)
Spring	17,873 (22.8%)	11,361 (22.8%)	5785 (22.6%)	727 (22.8%)
Summer	20,730 (26.4%)	12,930 (26.0%)	6972 (27.3%)	828 (25.9%)
Winter	16,620 (21.2%)	10,591 (21.3%)	5350 (20.9%)	679 (21.3%)
TVPA, mg, mean (SD)	38.4 (11.9)	39.7 (11.9)	36.5 (11.3)	32.0 (10.6)
MVPA, hour/day, mean (SD)	0.517 (0.447)	0.568 (0.462)	0.445 (0.410)	0.297 (0.340)
LPA, hour/day, mean (SD)	5.27 (1.58)	5.32 (1.56)	5.22 (1.59)	4.85 (1.70)
ST, hour/day, mean (SD)	7.98 (1.60)	7.97 (1.59)	7.98 (1.60)	8.07 (1.69)

*CVD *cardiovascular disease, *TVPA *total volume of physical activity, *MVPA *moderate-to-vigorous-intensity physical activity, *LPA *light-intensity physical activity, *ST *sedentary time, *mg *milligravity; health diet score was calculated based on self-reported servings of fruits, vegetables, whole grains, vegetable oil, fish, dairy, refined grains, unprocessed meats, processed meats, and sugar-sweetened beverages. More details can be found in Additional file 1: Table S4

Townsend Index (including measures of unemployment, non-car ownership, non-home ownership, and household overcrowding), derived from respondents’ postcode was used as an indicator of area-level SES

Employment status is categorized as employed (includes paid employment or self-employed, paid or voluntary work or student), retired, and inactive (includes looking after home and/or family, unable to work and unemployed)

Education is categorized as college or university degree, secondary school (includes A levels/AS levels or equivalent, O levels/GCSEs or equivalent, CSEs or equivalent), primary school, and professional qualification (NVQ or HND or HNC or equivalent, other professional qualifications)

### Joint associations of accelerometer-measured PA/ST and FI with all-cause mortality

**Fig. 1:**
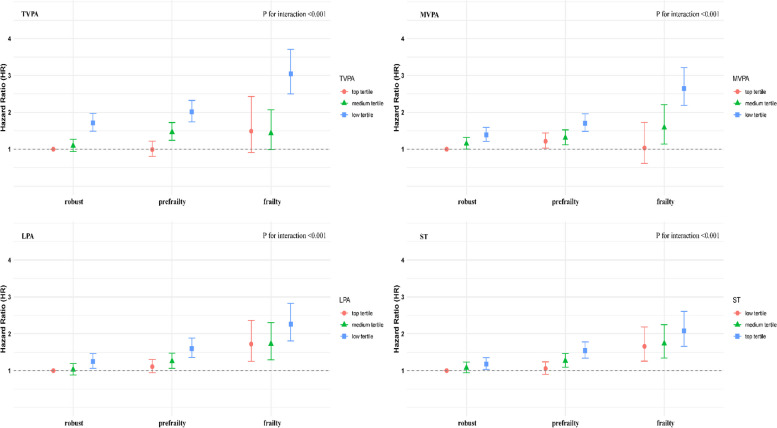
Joint associations of accelerometer-measured TVPA, MVPA, LPA, and ST with frailty status on all-cause mortality. TVPA, total volume of physical activity; MVPA, moderate-to-vigorous-intensity physical activity; LPA, light-intensity physical activity; ST, sedentary time; HR, hazard ratios; CI, confidence interval. Model adjusted for age at accelerometer measurement, sex, assessment center, body mass index, ethnicity, education, employment, household income, Townsend deprivation index, smoking status, alcohol drinking frequency, sleep duration, healthy diet score, family history of diabetes, family history of CVD, family history of cancer, seasonality, and total wear days. MVPA and LPA models were further adjusted for ST, the ST model was further adjusted for MVPA, while the TVPA model was not further adjusted

**Fig. 2:**
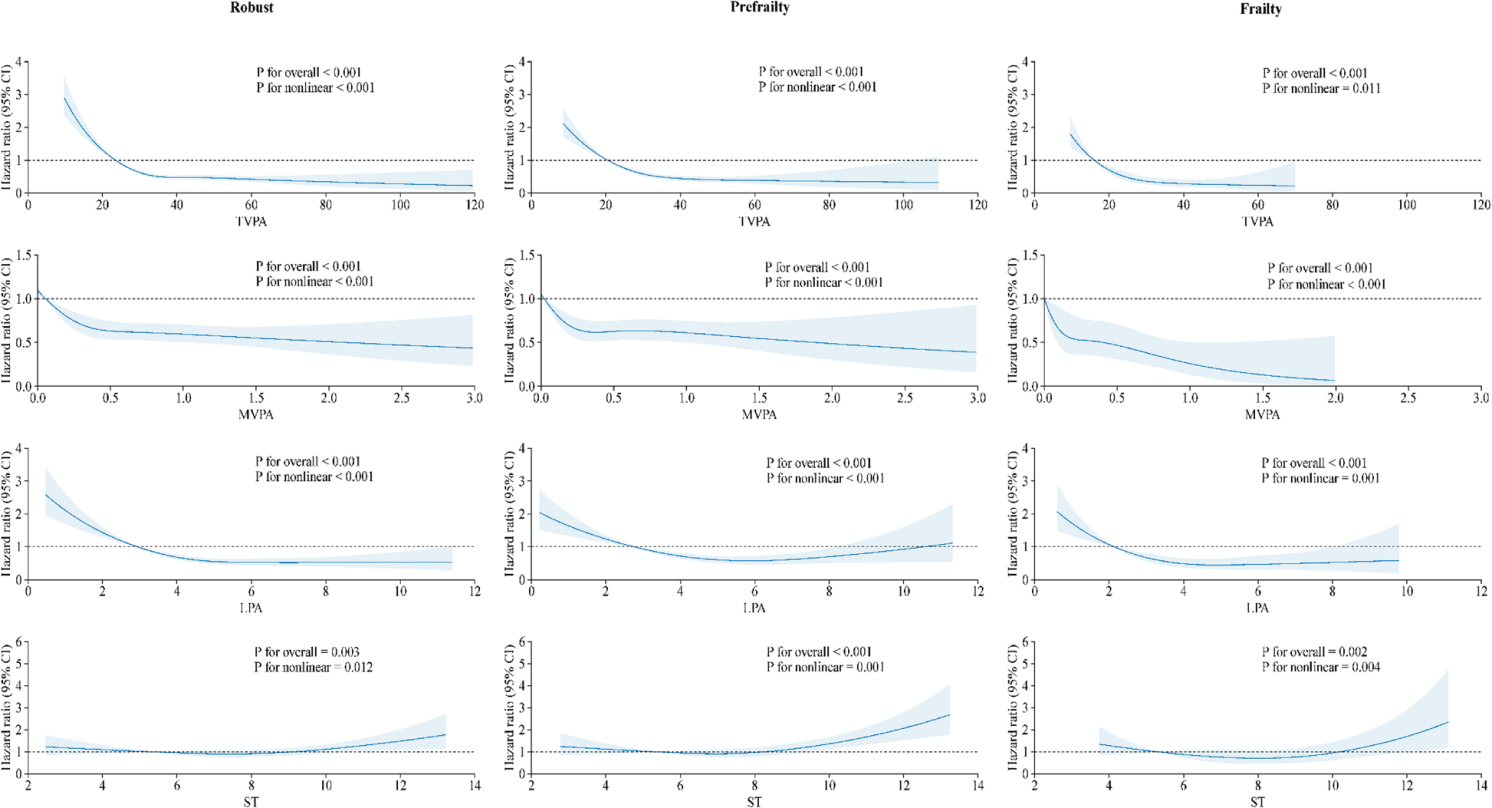
Dose–response associations between accelerometer-measured TVPA, MVPA, LPA, and ST with all-cause mortality by frailty index. TVPA, total volume of physical activity; MVPA, moderate-to-vigorous-intensity physical activity; LPA, light-intensity physical activity; ST, sedentary time; HR, hazard ratios; CI, confidence interval. Data were fitted by a restricted cubic spline Cox proportional hazards regression model, and the model was conducted with 4 knots at the 5th, 35th, 65th, and 95th percentiles of exposure (reference is the 5th percentile). Solid lines indicated HRs, and shadow shapes indicated 95% CIs. Model adjusted for age at accelerometer measurement, sex, assessment center, body mass index, ethnicity, education, employment, household income, Townsend deprivation index, smoking status, alcohol drinking frequency, sleep duration, healthy diet score, family history of diabetes, family history of CVD, family history of cancer, seasonality, and total wear days. MVPA and LPA models were further adjusted for ST, the ST model was further adjusted for MVPA, while the TVPA model was not further adjusted

The results of joint analyses involving accelerometer-measured PA/ST and the FI with all-cause mortality (Fig. 1 and Additional file 1: Table S11) revealed statistically significant interaction effects (all *P* for interaction < 0.05). The association between PA/ST and mortality was similar across all frailty categories. Specifically, within each frailty status, being in a higher tertile of TVPA, MVPA, and LPA was associated with a reduced risk of all-cause mortality, while being in a higher tertile of ST was associated with increased risk. Figure 2 shows the dose–response relationship of accelerometer-measured PA/ST with all-cause mortality stratified by FI categories. Dose–response curves, generated using restricted cubic splines, displayed similar and consistent non-linear patterns across FI categories (all *P* for nonlinear < 0.05). In terms of TVPA and MVPA, the HRs decreased progressively with increasing levels of PA, highlighting clear inverse associations. For LPA, following an initially steeper decline in HRs, there was an apparent flattening of the relationships. Regarding ST, after an initial slight decrease in HRs, there was a more pronounced upward trajectory.

### Life expectancy differences

**Fig. 3:**
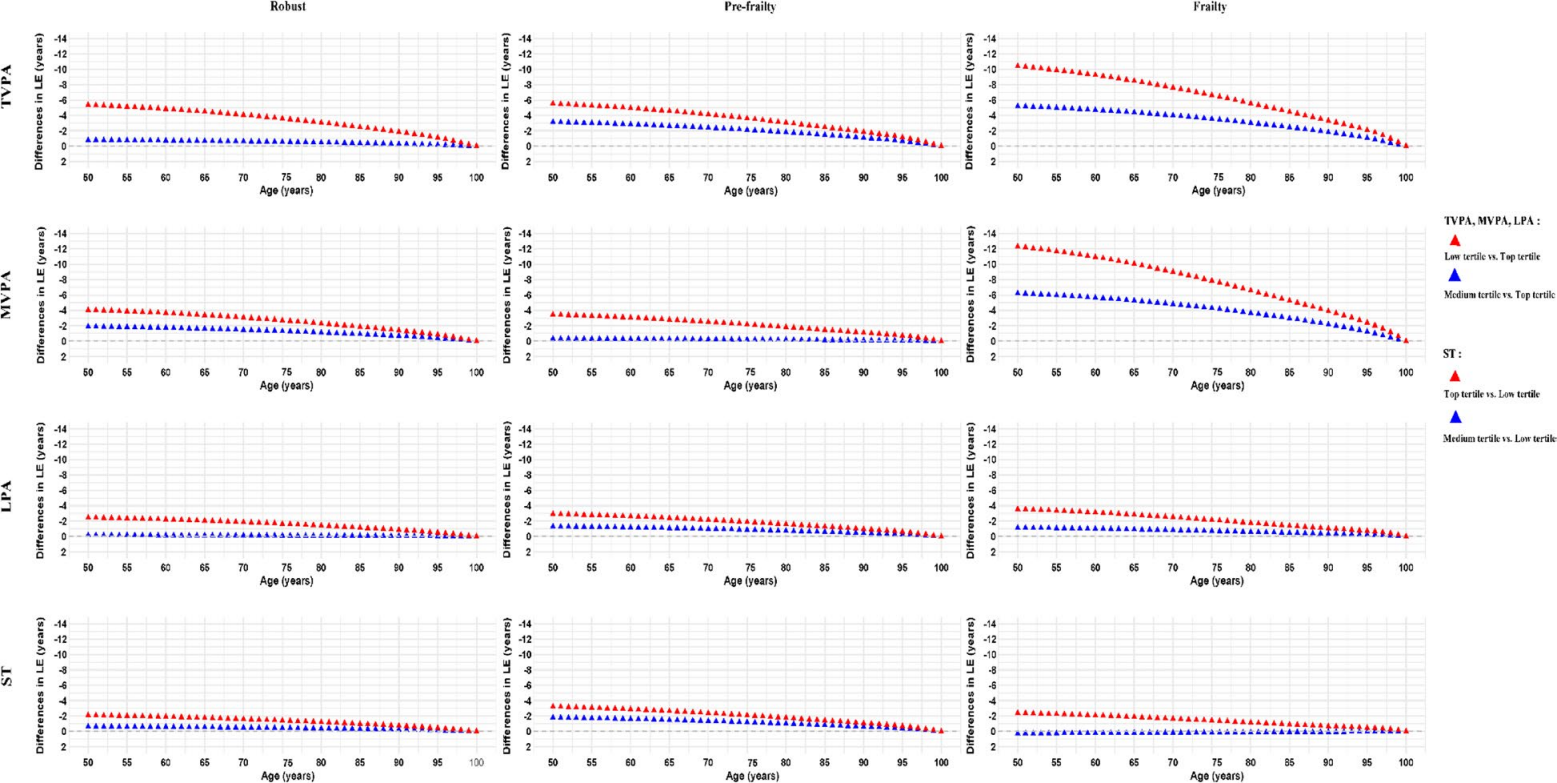
Differences in life expectancy in men by accelerometer-measured PA/ST across frailty index status. Lower life expectancy for TVPA, MVPA, and LPA was calculated by comparing the estimated life expectancy of individuals within the top tertile to those in the medium and low tertiles. Conversely, for ST, the calculation of reduced life expectancy was based on comparing individuals in the low tertile to those in the medium and top tertiles. TVPA, total volume of physical activity; MVPA, moderate-to-vigorous-intensity physical activity; LPA, light-intensity physical activity; ST, sedentary time; LE, life expectancy

Differences in estimated life expectancy from 50 years of age onward in men by accelerometer-measured PA/ST across FI status are presented in Fig. 3. Compared with the top tertile of TVPA, MVPA, and LPA, life expectancy was lower in the medium tertile and significantly more so for those in the low tertiles, a trend that persisted across frailty categories, being particularly pronounced in the frail group. Conversely, higher levels of ST were associated with shorter life expectancy, a pattern also consistent across different levels of frailty. At age 50, men with robust status in the lowest tertile of TVPA, MVPA, and LPA, or the highest tertile of ST, experienced reductions in life expectancy of 5.4 (95% CI, 4.8–6.0) years, 4.0 (95% CI, 3.0–5.0) years, 2.5 (95% CI, 1.8–3.2) years, and 2.1 (95% CI, 1.1–3.0) years, respectively, compared to their most physically active or least sedentary counterparts. More pronounced reductions were observed for men with frailty status, where life expectancy decreased by 10.4 (95% CI, 7.7–15.9) years, 12.3 (95% CI, 9.0–16.4) years, 3.6 (95% CI, 1.7–5.4) years, and 2.4 (95% CI, 1.3–3.5) years, respectively.

**Fig. 4:**
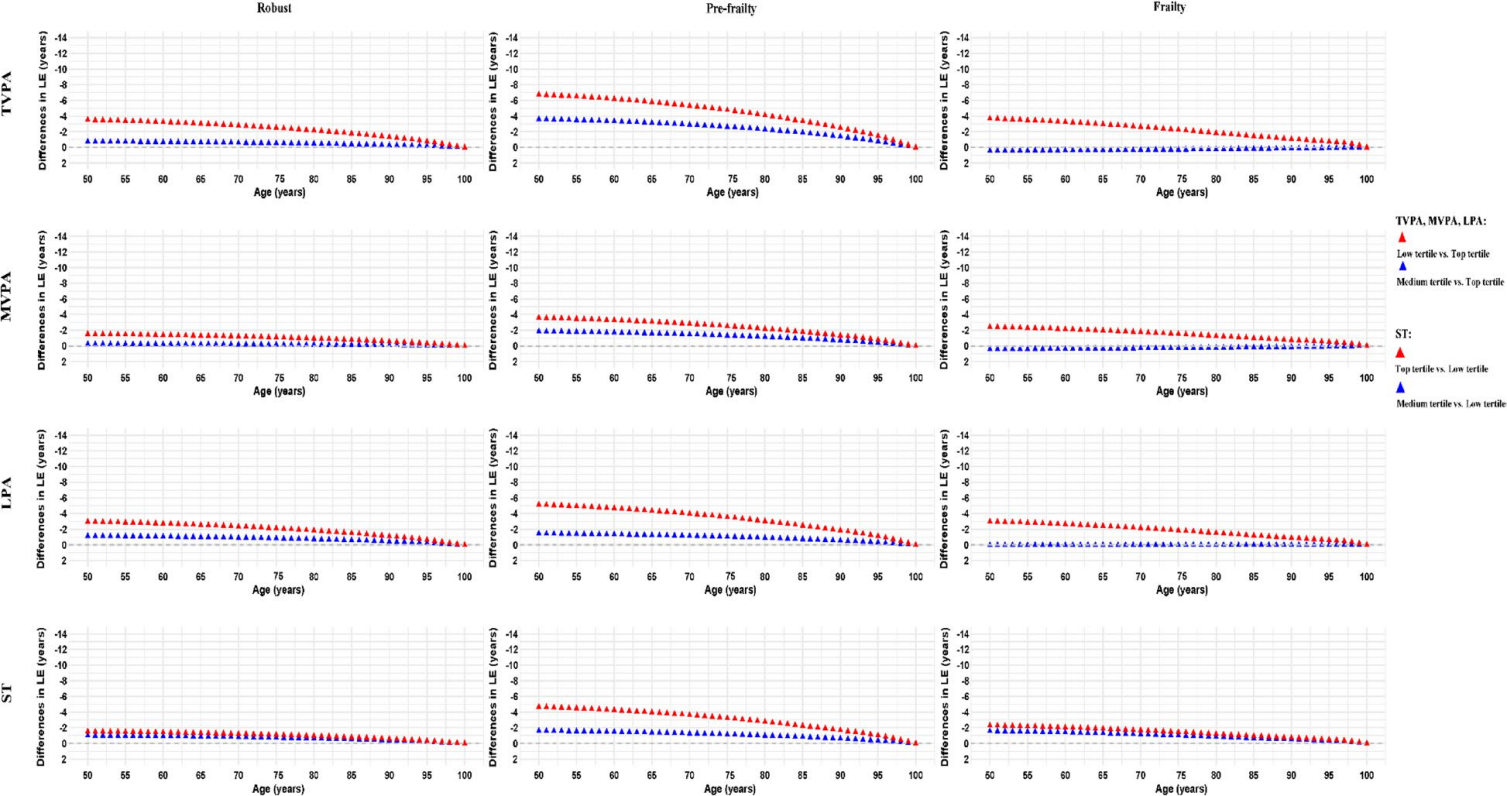
Differences in life expectancy in women by accelerometer-measured PA/ST across frailty index status. Lower life expectancy for TVPA, MVPA, and LPA was calculated by comparing the estimated life expectancy of individuals within the top tertile to those in the medium and low tertiles. Conversely, for ST, the calculation of reduced life expectancy was based on comparing individuals in the low tertile to those in the medium and top tertiles. TVPA, total volume of physical activity; MVPA, moderate-to-vigorous-intensity physical activity; LPA, light-intensity physical activity; ST, sedentary time; LE, life expectancy

Analogous to the patterns observed in men, women’s life expectancy decreased from the top to the medium tertile and even more so to the low tertile of TVPA, MVPA, and LPA, consistently across all frailty categories (Fig. 4). Likewise, higher ST was associated with a reduction in life expectancy, regardless of frailty categories. At age 50, robust women in the lowest tertile of TVPA, MVPA, and LPA or the highest tertile of ST had their life expectancy shortened by 3.5 (95% CI, 3.0–4.1) years, 1.5 (95% CI, 1.1–1.8) years, 3.0 (95% CI, 2.0–4.0) years, 1.5 (95% CI, 0.8–2.3) years, respectively, compared to their most physically active or least sedentary counterparts. Similarly, corresponding reductions in life expectancy were 3.7 (95% CI, 1.7–5.1), 2.4 (95% CI, 0.9–4.2), 3.0 (95% CI, 1.6–4.4), and 2.3 (95% CI, 0.8–3.9) years for frail women. Furthermore, we estimated life expectancy across age groups by FI in Additional file 1: Fig. S4 and by accelerometer-measured PA or ST in Additional file 1: Fig. S5.

### Sensitivity analyses

After excluding participants who died within the first 2 years of follow-up (Additional file 1: Table S12) or excluding all missing values for covariates (Additional file 1: Table S13), no substantial changes were observed in the associations with all-cause mortality. Similarly, excluding shift workers did not materially alter the results (Additional file 1: Table S14). Redefining awake times as 07:00 to 21:00 (Additional file 1: Fig. S4–S5, Table S15) and 08:00 to 20:00 (Additional file 1: Fig. S6–S7, Table S16) also had minimal impact on the estimates. Further mutual adjustment for MVPA and LPA did not materially influence the results (Additional file 1: Table S17). In addition, results regarding additive and multiplicative interactions between the accelerometer-measured PA/ST and the FI with all-cause mortality are displayed in Additional file 1: Table S18–S19. The independent associations of frailty index (Additional file 1: Table S20) and accelerometer-measured PA/ST (Additional file 1: Table S21) with all-cause mortality, as well as associations between covariates and frailty index (Additional file 1: Table S14) and covariates and all-cause mortality (Additional file 1: Table S15), were provided.

## Discussion

This large prospective study employed accelerometry to evaluate the combined effects of PA/ST and frailty status on all-cause mortality and life expectancy in middle-aged and older adults. Our analyses revealed that, within each frailty stratum, higher levels of TVPA, MVPA, and LPA were associated with lower mortality risk, whereas greater levels of ST with increased mortality risk. Dose–response trajectories revealed non-linear associations: inverse associations of greater TVPA, MVPA, and LPA levels with mortality risk, and positive association of higher ST level, remained consistent irrespective of frailty status. Remarkably, higher levels of TVPA, MVPA, and LPA, along with lower levels of ST, were associated with greater gains in life expectancy among frail individuals, with this pattern being more pronounced in men than in women.

Independent associations of accelerometer-measured PA/ST, and frailty status with all-cause mortality have been documented [5, 26–28], which was in line with our results. However, research on their combined effects remains limited. Our findings provided valuable insights into the associations of total PA volume and intensity-specific patterns with all-cause mortality across frailty categories, offering a more comprehensive understanding of these relationships. Specifically, dose–response analysis showed that higher levels of TVPA, reflecting a holistic measure of PA over waking hours, were associated with reduced mortality risk across frailty categories. This association exhibited no threshold effect, suggesting that encouraging greater overall movement volume may be a universal approach to support better health outcomes, regardless of frailty status. Notably, differences in the associations for MVPA and LPA were observed. Even after adjusting for ST or further mutual adjustments, MVPA demonstrated a clear and progressive inverse association with mortality, whereas LPA showed diminishing returns at higher levels, with risk reductions plateauing beyond a certain point. These observations aligned with prior evidence suggesting that MVPA were associated with greater metabolic and cardiovascular advantages and lower mortality risks, while LPA provided a more accessible option with relatively smaller but still meaningful associations with mortality risk [29, 30]. ST provided an important complementary perspective to these findings. Across all frailty categories, a higher level of ST was associated with greater mortality risk, with a clear upward trajectory in hazard ratios at higher levels. These associations persisted even when adjusted for MVPA, underscoring the significant and independent detrimental impact of prolonged ST on health. This reinforces the need for strategies that not only promote physical activity but also actively reduce ST to optimize health outcomes.

Our results supported a dual-focused strategy in health promotion. Increasing TVPA should remain a foundational goal for all individuals, as it captures the cumulative benefits of the overall movement. Public health efforts should prioritize MVPA for its superior health benefits, while recognizing LPA as a viable alternative for individuals with limited capacity for MVPA. Reducing ST must also be an integral part of health strategies, given its independent relationship with mortality risk. For frail individuals or those with physical limitations, interventions should focus on achievable increases in LPA and reductions in ST, acknowledging that even small improvements in LPA or decreases in ST are associated with better health outcomes.

An intriguing aspect of our study is the observed gender disparity in the associations between lower levels of TVPA, MVPA, and LPA, as well as higher levels of ST, with reduced life expectancy, with frail men being more affected than frail women. This phenomenon warrants further investigation into the underlying mechanisms. One possible explanation is that men, typically having higher muscle mass and oxygen uptake capacity, might experience a more significant impact from the decline in physical function than women as they age [31–33]. Additionally, men’s tendency towards more vigorous exercise could mean that any reduction in their activity levels might result in more pronounced changes relative to women [34, 35]. Moreover, men exhibit disproportionately higher mortality risks due to greater susceptibilities to conditions like cardiovascular disease, cancer, and metabolic disorders compared to women, which are among the primary drivers of life expectancy reductions [36].

Several mechanisms explaining the joint effects of PA/ST with FI on all-cause mortality have been explored. Frailty and metabolic syndrome (MetS) are intricately linked through mechanisms such as insulin resistance and chronic inflammation, which exacerbate the adverse effects of each other [37]. For example, in frail individuals, low PA and high ST intensify vulnerabilities, leading to endothelial dysfunction and arterial plaque buildup, heightening cardiovascular risks. Frailty often coexists with sarcopenia and reduced physical functioning, collectively accelerating the progression to diabetes and cardiovascular disease, worsening metabolic imbalances, and increasing the risk of complications such as kidney failure. Additionally, frailty exacerbates muscle dysfunction, intensified by low PA, leading to muscle atrophy and weakened respiratory muscles, which increases fall risks and susceptibility to respiratory diseases, potentially causing severe injuries or life-threatening complications, while compromised respiratory function further intensifies frailty and comorbidities, collectively elevating overall mortality risk [38]. Finally, frail individuals often have limited physiological reserves, and the combination of low PA and high ST could overwhelm their adaptive capacity, accelerating health deterioration and elevating the risk of mortality, potentially due to multi-organ failure [39].

It's important to note that both pre-frail and frail individuals still have a reduced life expectancy compared to those in robust states, affecting both men and women. This emphasizes the need for interventions targeting frailty, aiming to reduce or delay its onset in the elderly population. These results are not merely a concern for individual health but have broader implications that extend to healthcare systems and societal structures. The quantifiable differences in mortality and life expectancy identified in our study serve as both an essential empirical basis for shaping targeted preventive measures and a crucial reference for the formulation of healthcare policies for frail individuals. These findings also highlight the need for future research to investigate the underlying mechanisms, further addressing the unique needs of this population.

Unlike most studies that focus on individuals aged 60 and above, our study includes both middle-aged and older adults, offering a broader perspective on the impact of accelerometer-measured PA/ST and frailty on all-cause mortality during the transition from middle age to later life. The use of accelerometers in this analysis largely mitigated the recall bias commonly occurring in the application of self-reported questionnaires [40]. Although the single-time-point measurement of PA failed to capture its long-term dynamics, prior analyses have indicated that PA patterns tend to remain relatively stable over time [41]. Importantly, accelerometers cannot reliably detect certain activities, such as stair climbing, bicycling, or non-ambulatory muscle-strengthening exercises, potentially underestimating their contribution to overall physical activity. In defining the awake period as 06:00–22:00, we may not fully account for individuals with non-standard work or sleep patterns; however, sensitivity analyses excluding shift workers and using alternative awake windows (07:00–21:00 and 08:00–20:00) demonstrated that our findings were robust. Despite careful consideration of a wide range of confounding factors, covariates were sourced from interviews closest to the accelerometry date. This approach, while ensuring temporal proximity, may introduce subtle biases due to data availability across participants, but it remained widely employed in epidemiological research [21, 22]. Additionally, the observational study design inherently limited causal inference, though multiple sensitivity analyses supported the robustness of our results. The 49-item FI treats all deficits equally, not accounting for the varying severity of different health conditions, which could lead to individuals with the same number of deficits but differing in type and severity being classified into the same frailty category, potentially influencing the associations. The UK Biobank sample is not fully representative of the UK population, constraining the generalizability of our conclusions, yet evidence suggests that observed exposure-outcome associations are generally consistent with more representative samples [42]. Finally, despite the large overall sample size, certain stratified or interactive subgroups may have been underpowered by relatively small size, potentially affecting the precision of the estimates.

## Conclusions

In conclusion, our study demonstrated that greater PA volume and increased engagement in intensity-specific patterns, including MVPA and LPA, alongside reduced ST, were associated with substantially lower mortality risk and longer life expectancy across the frailty spectrum.

## Supplementary Information

Additional file 1. Fig. S1. Flowchart of participant enrolment. Fig. S2. Correlation between accelerometer-measured PA and ST. Fig. S3. Timeline of some covariates collection. Fig. S4. The estimates of cumulative survival time from 50 years of age onward among women and men among different levels of frailty index. Fig. S5. The estimates of cumulative survival time from 50 years of age onward among women and men among different levels of accelerometer-measured PA and ST. Fig. S6. Joint associations of accelerometer-measured TVPA, MVPA, LPA and ST with all-cause mortality (awake time: 07:00–21:00). Fig. S7. Dose–response associations (HR and 95%CI) between accelerometer-measured TVPA, MVPA, LPA, ST with all-cause mortality by frailty index categories using restricted cubic splines with four knots located at the 5th, 35th, 65th, 95th percentiles of each exposure (awake time: 07:00–21:00). Fig. S8. Joint associations of accelerometer-measured TVPA, MVPA, LPA and ST with all-cause mortality (awake time: 08:00–20:00). Fig. S9. Dose–response associations (HR and 95%CI) between accelerometer-measured TVPA, MVPA, LPA, ST with all-cause mortality by frailty index categories using restricted cubic splines with four knots located at the 5th, 35th, 65th, and 95th percentiles of each exposure (awake time: 08:00–20:00). Table S1. Comparison of baseline characteristics of participants with and without complete frailty index data. Table S2. Items used for constructing frailty index. Table S3. The resource and definition of the covariates. Table S4. Definition of each component of a healthy diet score. Table S5. The numbers (percentages) of participants with missing covariates. Table S6. Baseline characteristics of the participants stratified by frailty index (before imputation). Table S7. Baseline characteristics of the participants stratified by TVPA. Table S8. Baseline characteristics of the participants stratified by MVPA. Table S9. Baseline characteristics of the participants stratified by LPA. Table S10. Baseline characteristics of the participants stratified by ST. Table S11. Joint associations of accelerometer-measured TVPA, MVPA, LPA, and ST with frailty status on all-cause mortality. Table S12. Joint associations of accelerometer-measured TVPA, MVPA, LPA, and ST with frailty status on all-cause mortality (remove deaths within first 2 years, *n* = 78,062). Table S13. Joint associations of accelerometer-measured TVPA, MVPA, LPA, and ST with frailty status on all-cause mortality (remove missing values, *n* = 64,771). Table S14. Joint associations of accelerometer-measured TVPA, MVPA, LPA, and ST with frailty status on all-cause mortality (remove night shift workers, *n* = 75,571). Table S15. Joint associations of accelerometer-measured TVPA, MVPA, LPA, and ST with frailty status on all-cause mortality (awake time: 07:00–21:00). Table S16. Joint associations of accelerometer-measured TVPA, MVPA, LPA, and ST with frailty status on all-cause mortality (awake time: 08:00–20:00). Table S17. Joint associations of accelerometer-measured MVPA and LPA with frailty status on all-cause mortality (further mutually adjusted for MVPA or LPA). Table S18. Analyses on interaction of accelerometer-measured physical activity and sedentary and pre-frailty with all-cause mortality. Table S19. Analyses on interaction of accelerometer-measured physical activity and sedentary and frailty with all-cause mortality. Table S20. Association between frailty index and all-cause mortality. Table S21. Association between accelerometer-measured TVPA, MVPA, LPA, and ST and all-cause mortality. Table S22. Association between various covariates and frailty index using logistic regression models (reference group: robust). Table S23. Association between various covariates and all-cause mortality using cox regression models. Supplementary Method. Estimating the differences in life expectancy.

## Data Availability

The dataset supporting the conclusions of this article is available on the website: https://www.ukbiobank.ac.uk/. Access to the UK Biobank resource can be obtained via an approved application.

## Abbreviations

FI: Frailty index
PA: Physical activity
TVPA: Total volume of physical activity
MVPA: Moderate-to-vigorous-intensity physical activity
LPA: Light-intensity physical activity
ST: Sedentary time
HR: Hazard ratio
CI: Confidence interval
ENMO: Euclidean Norm Minus One
mg: Milli-gravity
METs: Metabolic equivalent of tasks
BMI: Body mass index
NHS: National Health Service
SD: Standard deviations
CVD: Cardiovascular disease
MetS: Metabolic syndrome
