# Supplementary material for: Accelerometer-measured physical activity, frailty, and all-cause mortality and life expectancy among middle-aged and older adults: a UK Biobank longitudinal study
Source: BMC Med. 2025 Feb 27;23:125. doi: 10.1186/s12916-025-03960-z (PMC11866850; doi:10.1186/s12916-025-03960-z)
Supplement: Supplementary file 1 — Additional file 1. Fig. S1. Flowchart of participant enrolment. Fig. S2. Correlation between accelerometer-measured PA and ST. Fig. S3. Timeline of some covariates collection. Fig. S4. The estimates of cumulative survival time from 50 years of age onward among women and men among different levels of frailty index. Fig. S5. The estimates of cumulative survival time from 50 years of age onward among women and men among different levels of accelerometer-measured PA and ST. Fig. S6. Joint associations of accelerometer-measured TVPA, MVPA, LPA and ST with all-cause mortality (awake time: 07:00–21:00). Fig. S7. Dose–response associations (HR and 95%CI) between accelerometer-measured TVPA, MVPA, LPA, ST with all-cause mortality by frailty index categories using restricted cubic splines with four knots located at the 5th, 35th, 65th, 95th percentiles of each exposure (awake time: 07:00–21:00). Fig. S8. Joint associations of accelerometer-measured TVPA, MVPA, LPA and ST with all-cause mortality (awake time: 08:00–20:00). Fig. S9. Dose–response associations (HR and 95%CI) between accelerometer-measured TVPA, MVPA, LPA, ST with all-cause mortality by frailty index categories using restricted cubic splines with four knots located at the 5th, 35th, 65th, and 95th percentiles of each exposure (awake time: 08:00–20:00). Table S1. Comparison of baseline characteristics of participants with and without complete frailty index data. Table S2. Items used for constructing frailty index. Table S3. The resource and definition of the covariates. Table S4. Definition of each component of a healthy diet score. Table S5. The numbers (percentages) of participants with missing covariates. Table S6. Baseline characteristics of the participants stratified by frailty index (before imputation). Table S7. Baseline characteristics of the participants stratified by TVPA. Table S8. Baseline characteristics of the participants stratified by MVPA. Table S9. Baseline characteristics of the participants st [file 12916_2025_3960_MOESM1_ESM.docx]

**Additional file 1**

**Accelerometer-measured physical activity, frailty, and all-cause mortality and life expectancy among middle-aged and older adults: a UK Biobank longitudinal study**

**Fig. S1.** Flowchart of participant enrolment

**Fig. S2.** Correlation between accelerometer-measured PA and ST

**Fig. S3.** Timeline of some covariates collection

**Fig. S4.** The estimates of cumulative survival time from 50 years of age onward among women and men among different levels of frailty index

**Fig. S5.** The estimates of cumulative survival time from 50 years of age onward among women and men among different levels of accelerometer-measured PA and ST

**Fig. S6.** Joint associations of accelerometer-measured TVPA, MVPA, LPA and ST with all-cause mortality (awake time: 07:00-21:00)

**Fig. S7.** Dose-response associations (HR and 95%CI) between accelerometer-measured TVPA, MVPA, LPA, ST with all-cause mortality by frailty index categories using restricted cubic splines with four knots located at the 5th, 35th, 65th, 95th percentiles of each exposure (awake time: 07:00-21:00)

**Fig. S8.** Joint associations of accelerometer-measured TVPA, MVPA, LPA and ST with all-cause mortality (awake time: 08:00-20:00)

**Fig. S9.** Dose-response associations (HR and 95%CI) between accelerometer-measured TVPA, MVPA, LPA, ST with all-cause mortality by frailty index categories using restricted cubic splines with four knots located at the 5th, 35th, 65th, and 95th percentiles of each exposure (awake time: 08:00-20:00)

**Table S1.** Comparison of baseline characteristics of participants with and without complete frailty index data

**Table S2.** Items used for constructing frailty index

**Table S3.** The resource and definition of the covariates

**Table S4.** Definition of each component of a healthy diet score

**Table S5.** The numbers (percentages) of participants with missing covariates

**Table S6.** Baseline characteristics of the participants stratified by frailty index (before imputation)

**Table S7.** Baseline characteristics of the participants stratified by TVPA

**Table S8.** Baseline characteristics of the participants stratified by MVPA

**Table S9.** Baseline characteristics of the participants stratified by LPA

**Table S10.** Baseline characteristics of the participants stratified by ST

**Table S11.** Joint associations of accelerometer-measured TVPA, MVPA, LPA, and ST with frailty status on all-cause mortality

**Table S12.** Joint associations of accelerometer-measured TVPA, MVPA, LPA, and ST with frailty status on all-cause mortality (remove deaths within first 2 years, n=78062)

**Table S13.** Joint associations of accelerometer-measured TVPA, MVPA, LPA, and ST with frailty status on all-cause mortality (remove missing values, n=64771)

**Table S14.** Joint associations of accelerometer-measured TVPA, MVPA, LPA, and ST with frailty status on all-cause mortality (remove night shift workers, n=75571)

**Table S15.** Joint associations of accelerometer-measured TVPA, MVPA, LPA, and ST with frailty status on all-cause mortality (awake time: 07:00-21:00)

**Table S16.** Joint associations of accelerometer-measured TVPA, MVPA, LPA, and ST with frailty status on all-cause mortality (awake time: 08:00-20:00)

**Table S17.** Joint associations of accelerometer-measured MVPA and LPA with frailty status on all-cause mortality (further mutually adjusted for MVPA or LPA)

**Table S18.** Analyses on interaction of accelerometer-measured physical activity and sedentary and pre-frailty with all-cause mortality

**Table S19.** Analyses on interaction of accelerometer-measured physical activity and sedentary and frailty with all-cause mortality

**Table S20.** Association between frailty index and all-cause mortality

**Table S21.** Association between accelerometer-measured TVPA, MVPA, LPA, and ST and all-cause mortality

**Table S22.** Association between various covariates and frailty index using logistic regression models (reference group: robust)

**Table S23.** Association between various covariates and all-cause mortality using cox regression models

**Supplementary Method.** Estimating the differences in life expectancy

**Fig. S1. Flowchart of participant enrolment**

502,389 participants recruited to the UK Biobank at the baseline

398,962 participants were excluded:

- 117 withdrew from UK Biobank
- 398,845 did not participate in the accelerometer assessment

103,427 participants had accelerometer data

11,243 participants were excluded:

- 2,592 could not be calibrated
- 8,638 did not wear the device for sufficiently long time
- 13 had implausibly high values (average ENMO > 100 mg)

92,184 participants had valid accelerometer data

13,483 participants without complete data for constructing 49-item frailty index

78,508 participants included in the main analysis (impute data for 13,737 with missing covariates)

5 participants were excluded due to incomplete data for outcome

188 participants with age at accelerometer measurement < 45 years were excluded

ENMO: Euclidean Norm Minus One (ENMO) in milli-gravity (mg) was used to measure movement-related acceleration.

**Fig. S2. Correlation between accelerometer-measured PA and ST**


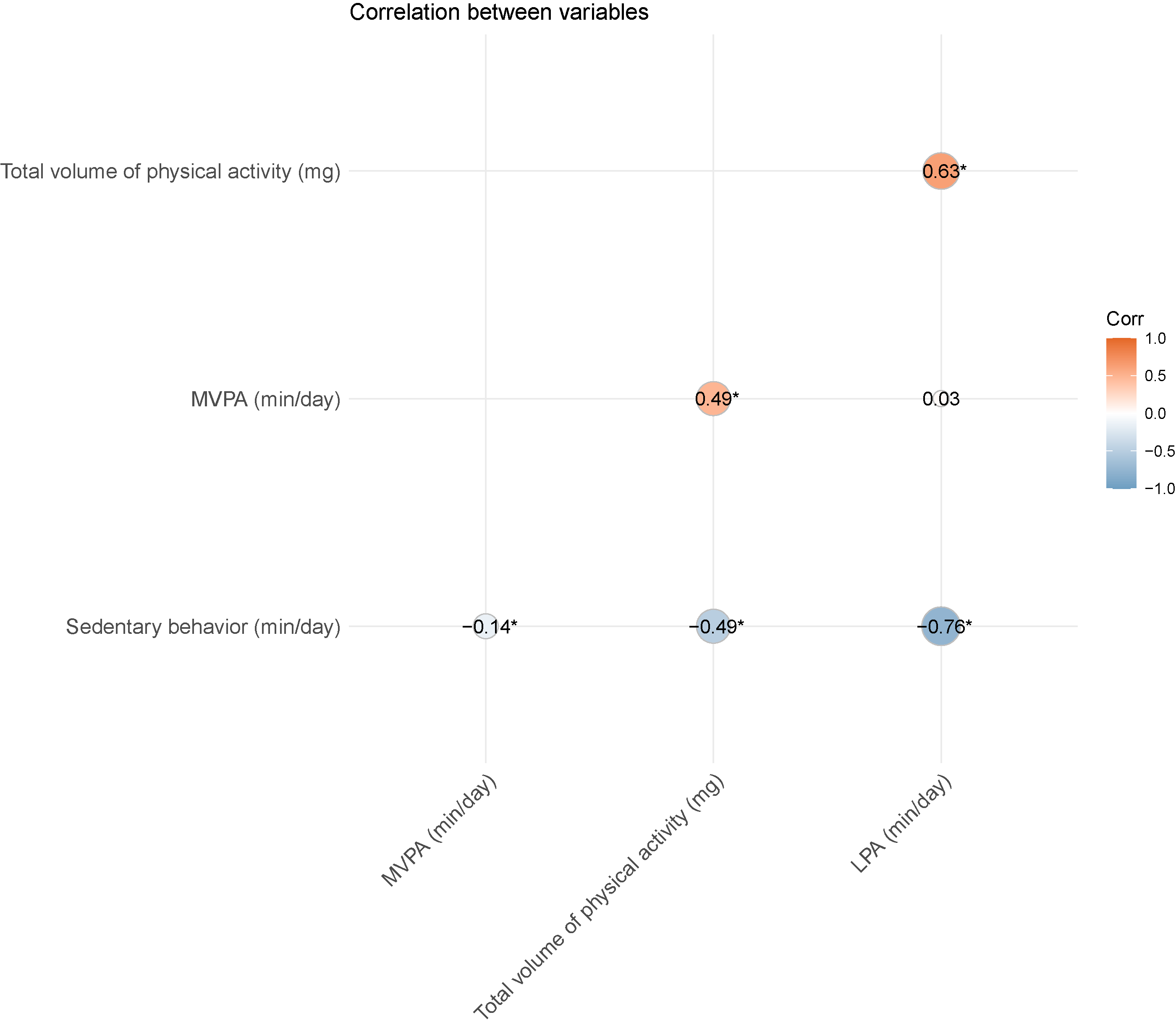


Spearman correlation analysis was performed. * p value <0.05.

**Fig. S3. Timeline of some covariates collection**


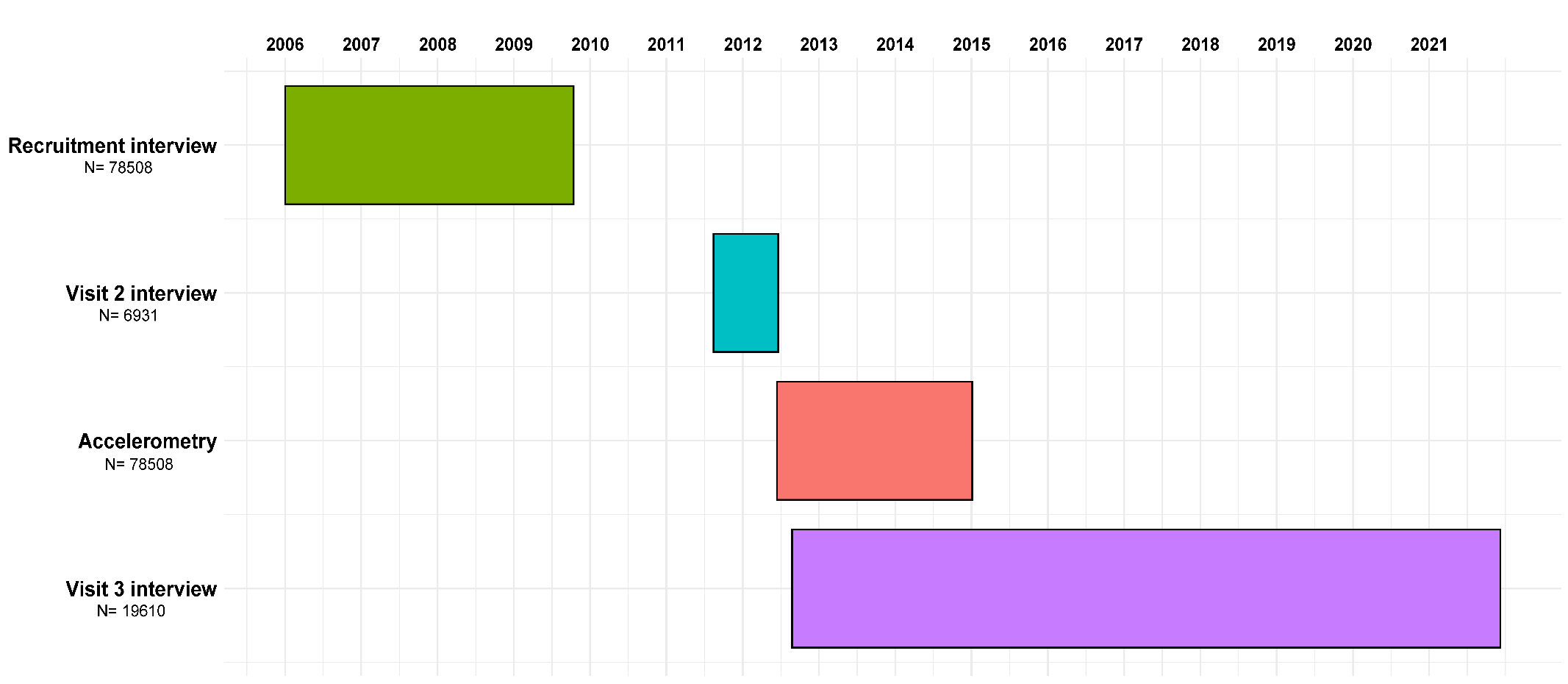


The covariates with repeated measurements include education level, employment status, household income, smoking status, alcohol consumption, healthy diet score, sleep duration, and body mass index (BMI) were obtained from touchscreen questionnaires at the time-point closet to the accelerometry.

**Fig. S4. The estimates of cumulative survival time from 50 years of age onward among women and men among different levels of frailty index**

**
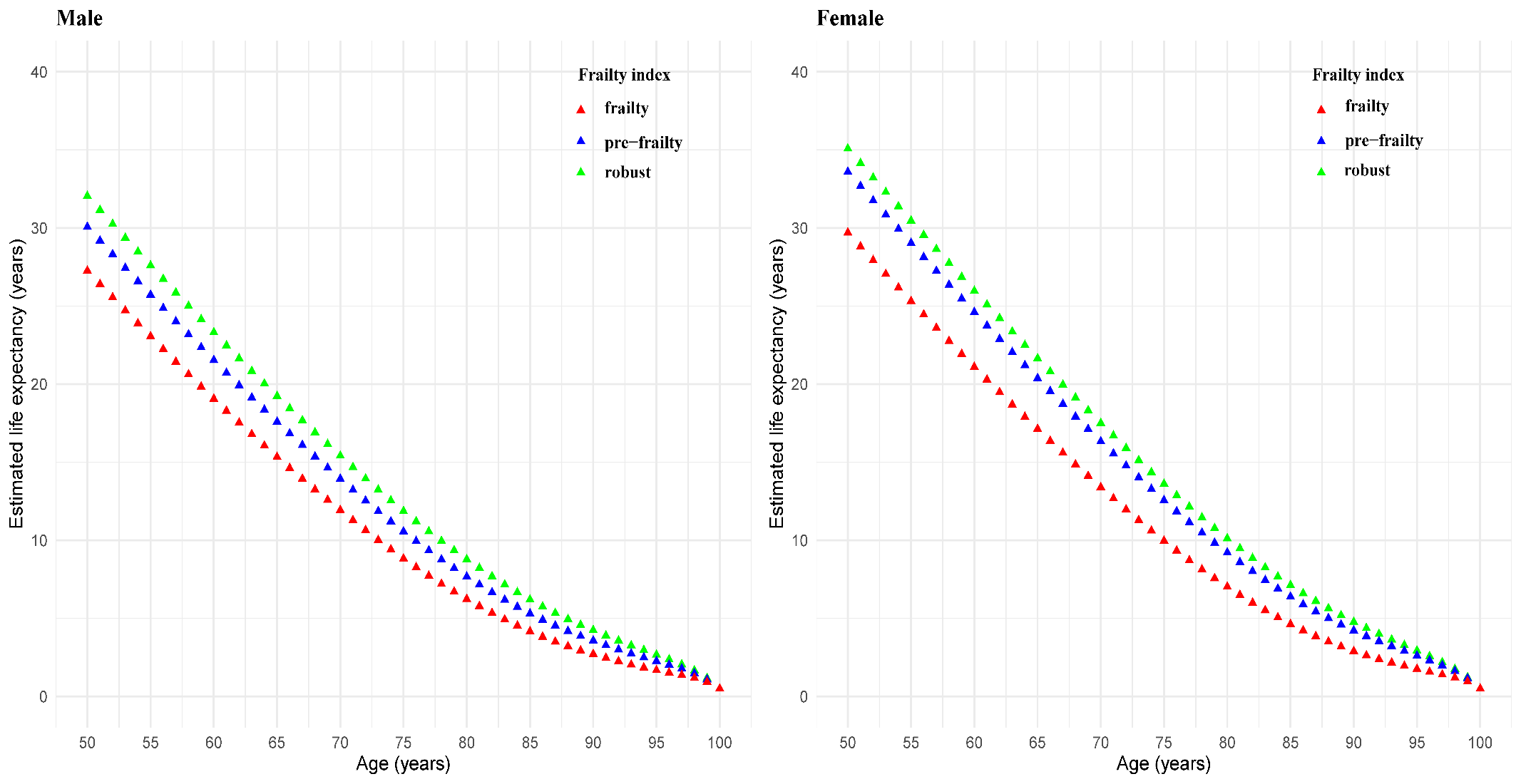
**

**Fig. S5. The estimates of cumulative survival time from 50 years of age onward among women and men among different levels of accelerometer-measured PA and ST**

**
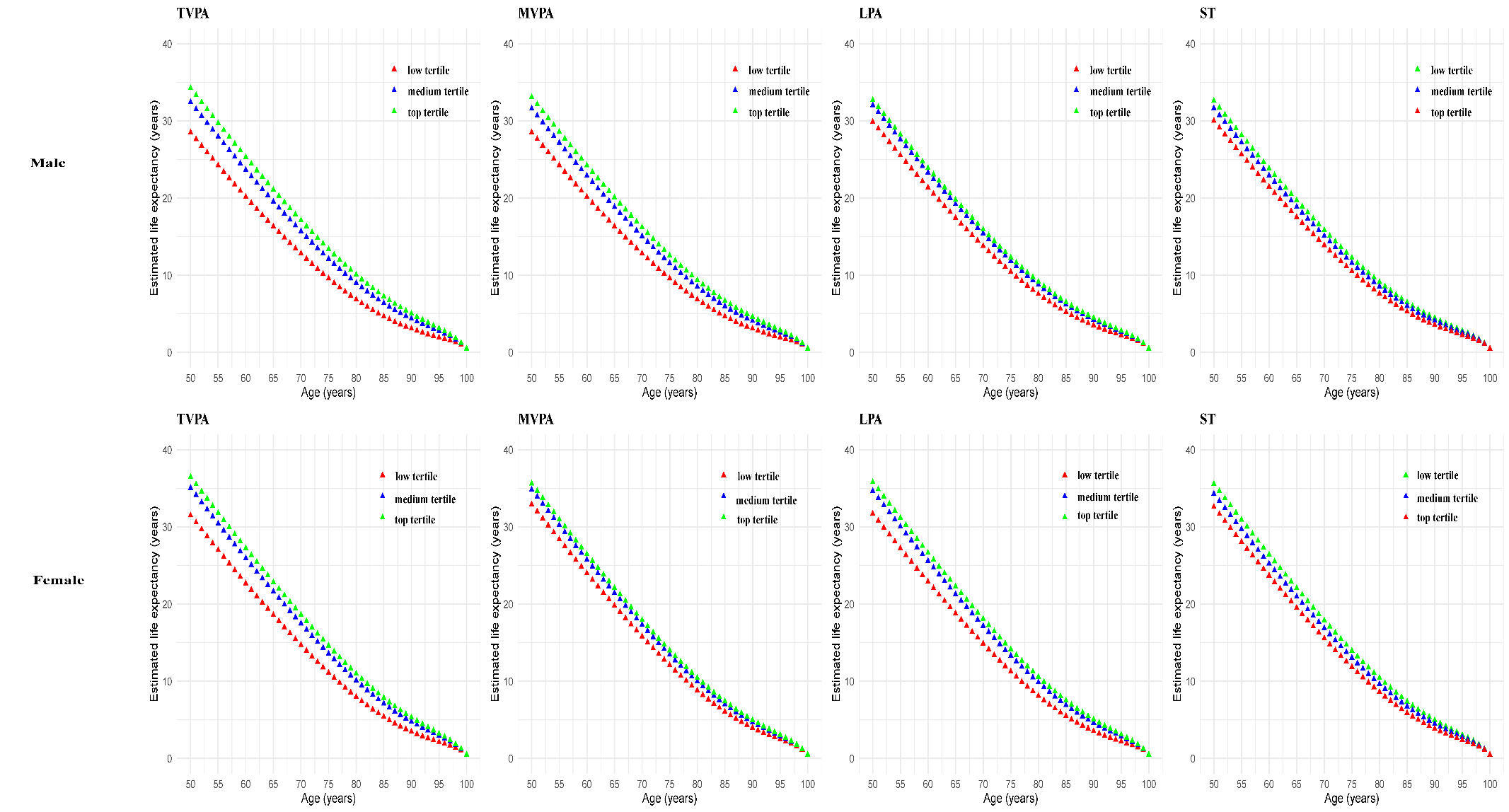
**

**Fig. S6. Joint associations of accelerometer-measured TVPA, MVPA, LPA and ST with all-cause mortality (awake time: 07:00-21:00)**

**
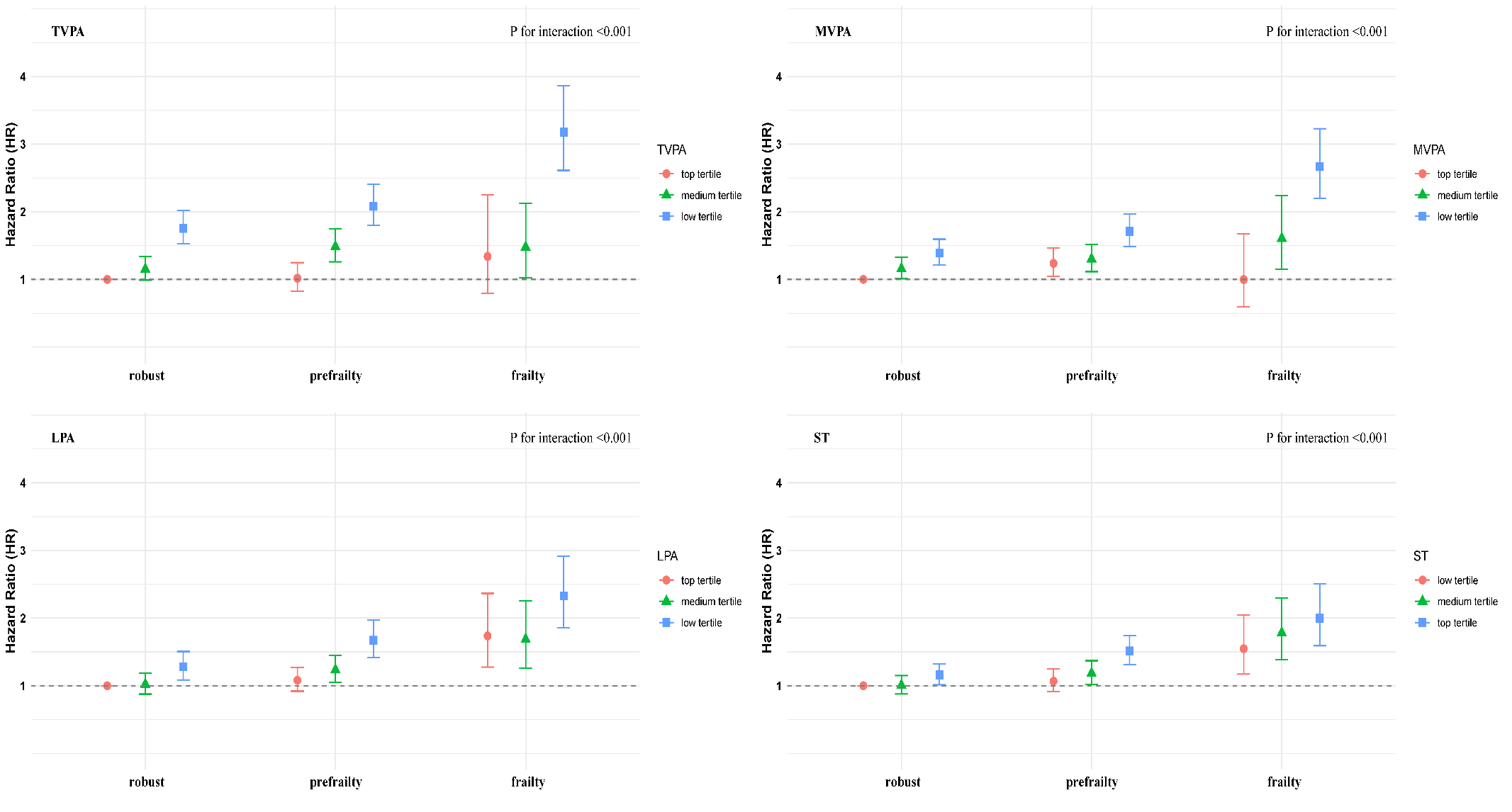
**

TVPA, total volume of physical activity; MVPA, moderate- to- vigorous- intensity physical activity; LPA, light- intensity physical activity; ST, sedentary time; HR, hazard ratios; CI, confidence interval.

Model adjusted for age at accelerometer measurement, sex, assessment center, body mass index, ethnicity, education, employment, household income, Townsend deprivation index, smoking status, alcohol drinking frequency, sleep duration, healthy diet score, family history of diabetes, family history of CVD, family history of cancer, seasonality, and total wear days. MVPA and LPA models were further adjusted for ST, ST model was further adjusted for MVPA, while TVPA model was not further adjusted.

**Fig. S7. Dose-response associations (HR and 95%CI) between accelerometer-measured TVPA, MVPA, LPA, ST with all-cause mortality by frailty index categories using restricted cubic splines with four knots located at the 5th, 35th, 65th, 95th percentiles of each exposure (awake time: 07:00-21:00)**

**
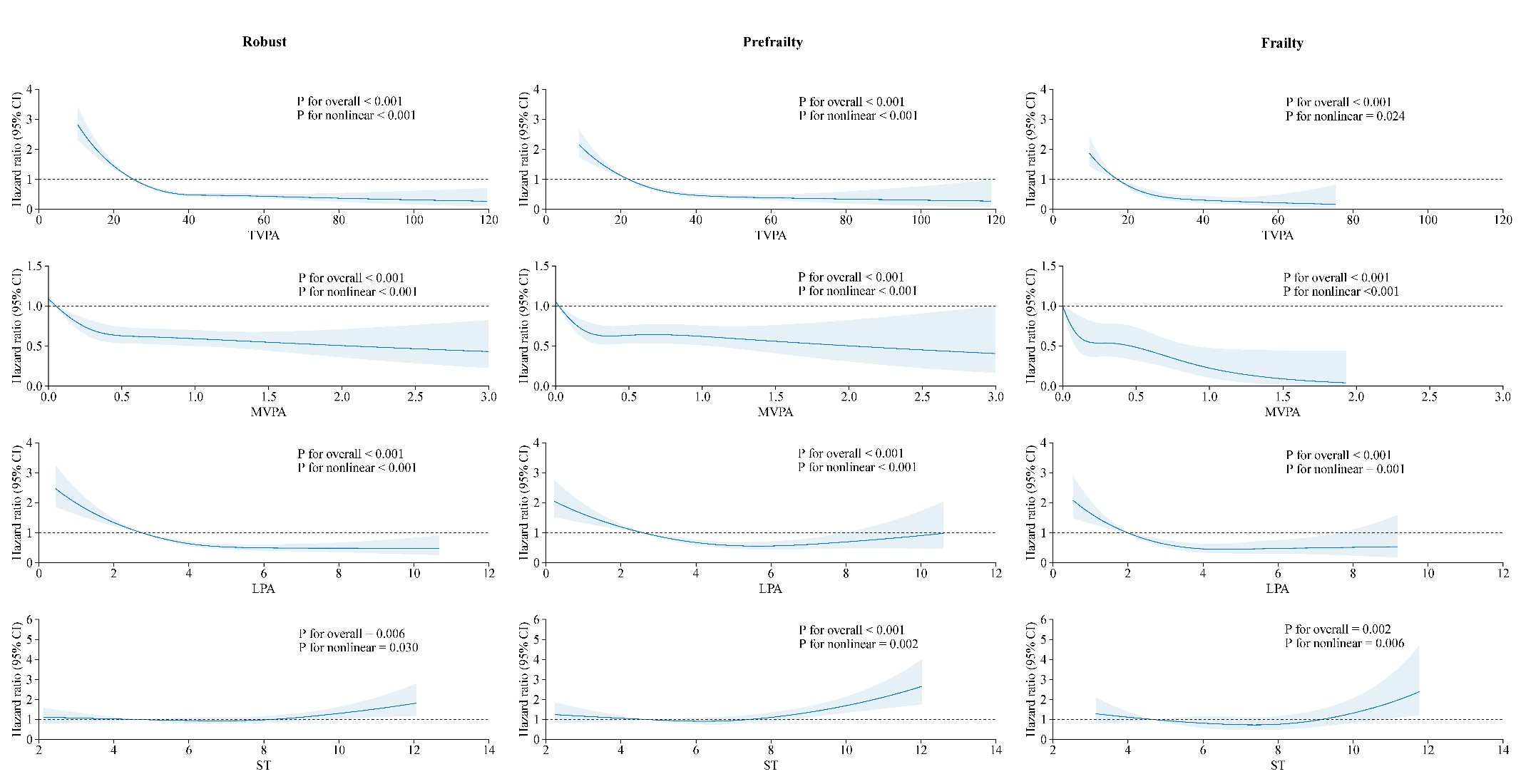
**

TVPA, total volume of physical activity; MVPA, moderate- to- vigorous- intensity physical activity; LPA, light- intensity physical activity; ST, sedentary time; HR, hazard ratios; CI, confidence interval.

Data were fitted by a restricted cubic spline Cox proportional hazards regression model, and the model was conducted with 4 knots at the 5th, 35th, 65th, 95th percentiles of exposure (reference is the 5th percentile). Solid lines indicated HRs, and shadow shape indicated 95% CIs. Model adjusted for age at accelerometer measurement, sex, assessment center, body mass index, ethnicity, education, employment, household income, Townsend deprivation index, smoking status, alcohol drinking frequency, sleep duration, healthy diet score, family history of diabetes, family history of CVD, family history of cancer, seasonality, and total wear days. MVPA and LPA models were further adjusted for ST, ST model was further adjusted for MVPA, while TVPA model was not further adjusted.

**Fig. S8. Joint associations of accelerometer-measured TVPA, MVPA, LPA and ST with all-cause mortality (awake time: 08:00-20:00)**

**
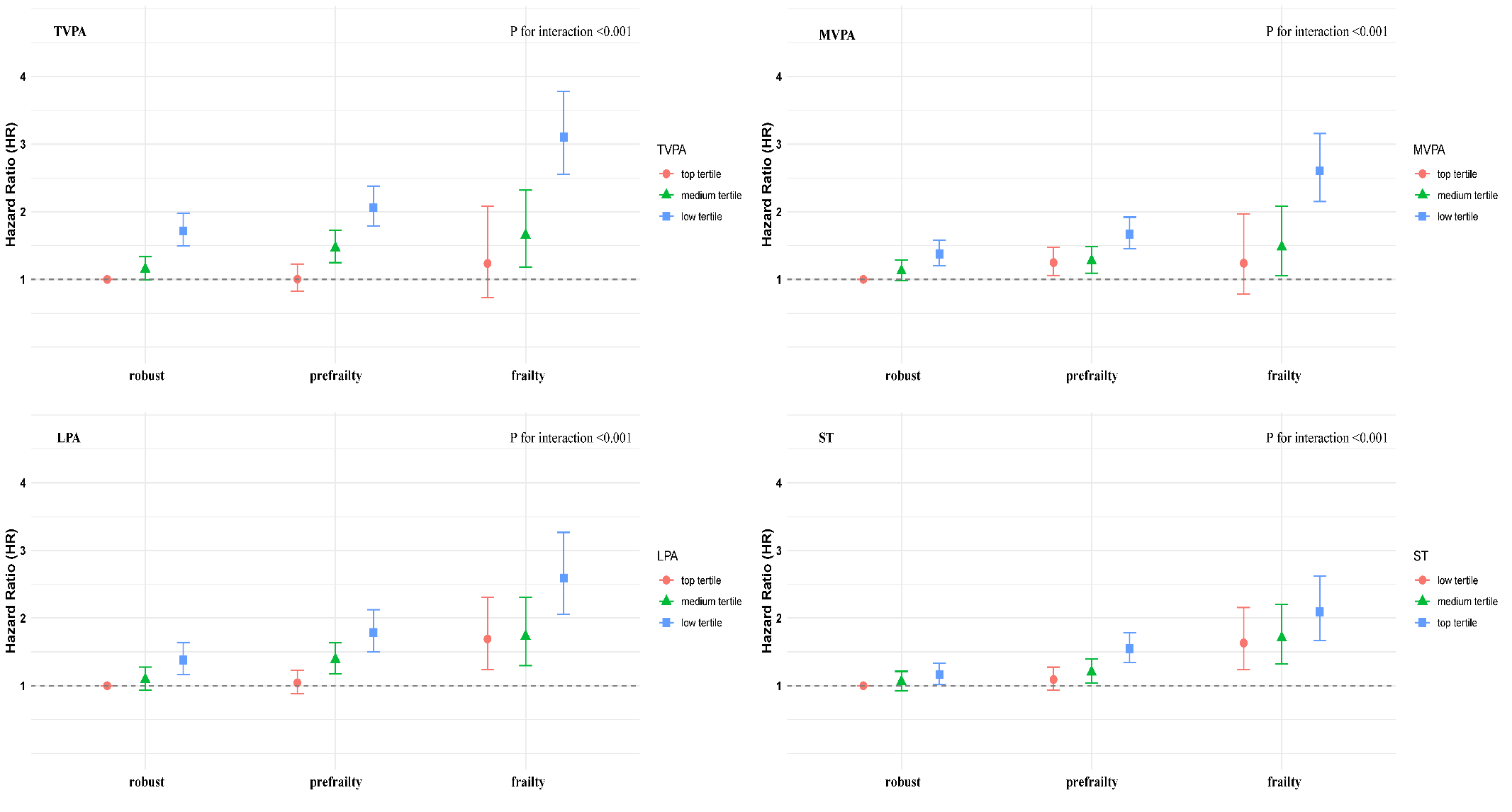
**

TVPA, total volume of physical activity; MVPA, moderate- to- vigorous- intensity physical activity; LPA, light- intensity physical activity; ST, sedentary time; HR, hazard ratios; CI, confidence interval.

Model adjusted for age at accelerometer measurement, sex, assessment center, body mass index, ethnicity, education, employment, household income, Townsend deprivation index, smoking status, alcohol drinking frequency, sleep duration, healthy diet score, family history of diabetes, family history of CVD, family history of cancer, seasonality, and total wear days. MVPA and LPA models were further adjusted for ST, ST model was further adjusted for MVPA, while TVPA model was not further adjusted.

**Fig. S9. Dose-response associations (HR and 95%CI) between accelerometer-measured TVPA, MVPA, LPA, ST with all-cause mortality by frailty index categories using restricted cubic splines with four knots located at the 5th, 35th, 65th, and 95th percentiles of each exposure (awake time: 08:00-20:00)**

**
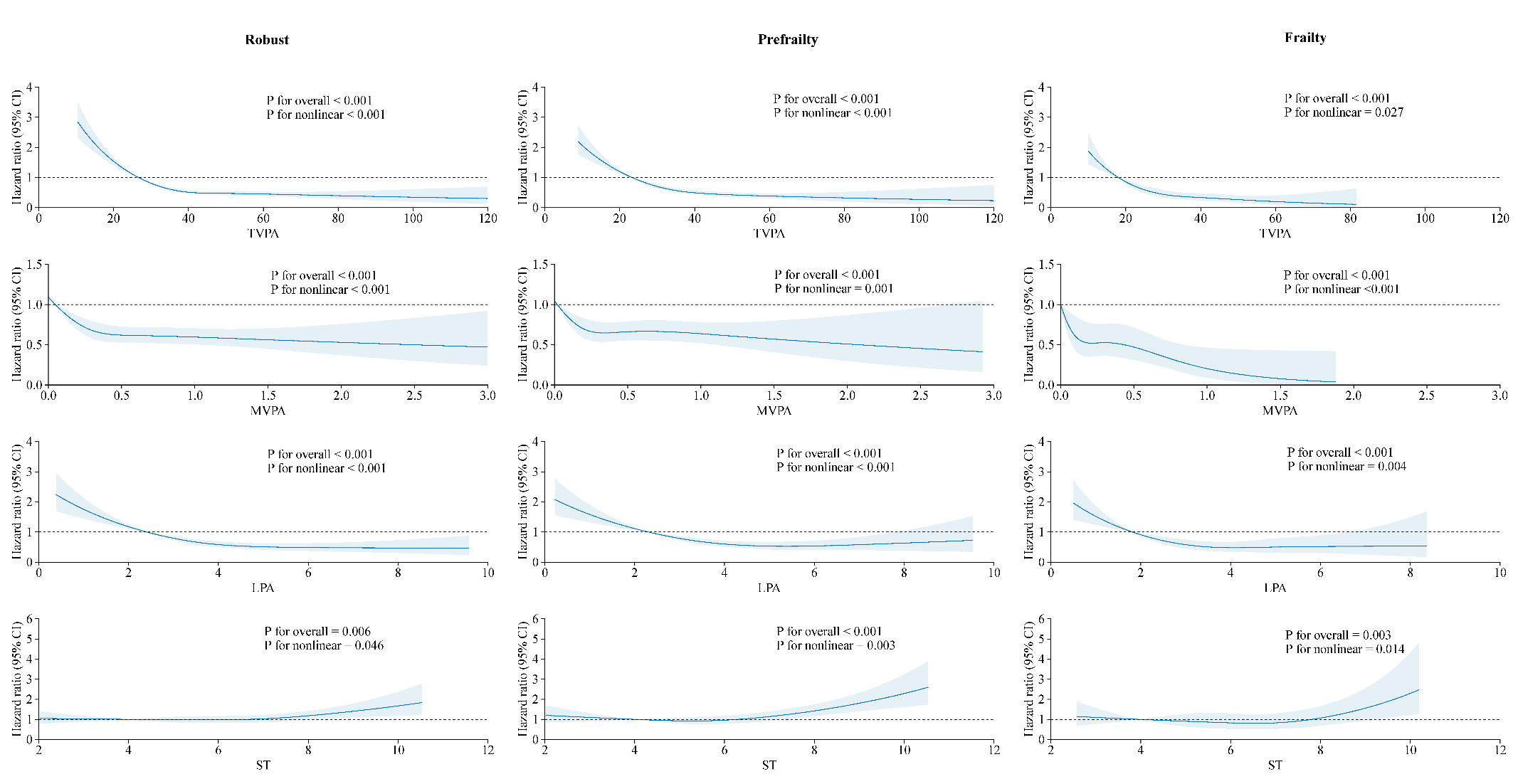
**

TVPA, total volume of physical activity; MVPA, moderate- to- vigorous- intensity physical activity; LPA, light- intensity physical activity; ST, sedentary time; HR, hazard ratios; CI, confidence interval.

Data were fitted by a restricted cubic spline Cox proportional hazards regression model, and the model was conducted with 4 knots at the 5th, 35th, 65th, 95th percentiles of exposure (reference is the 5th percentile). Solid lines indicated HRs, and shadow shape indicated 95% CIs. Model adjusted for age at accelerometer measurement, sex, assessment center, body mass index, ethnicity, education, employment, household income, Townsend deprivation index, smoking status, alcohol drinking frequency, sleep duration, healthy diet score, family history of diabetes, family history of CVD, family history of cancer, seasonality, and total wear days. MVPA and LPA models were further adjusted for ST, ST model was further adjusted for MVPA, while TVPA model was not further adjusted.

**Table S1. Comparison of baseline characteristics of participants with and without complete frailty index data**

|  | **Without complete data** | **With complete data** |
| --- | --- | --- |
|  | (N=13472) | (N=78701) |
| Age at accelerometer measurement, mean (SD) | 62.2 (7.86) | 61.9 (7.84) |
| Sex, n (%) |  |  |
| female | 8260 (61.3%) | 43640 (55.5%) |
| male | 5212 (38.7%) | 35061 (44.5%) |
| Body mass index (BMI), kg/m^2^, n (%) |  |  |
| <25.0 | 5079 (37.7%) | 31221 (39.7%) |
| 25.0-29.9 | 5312 (39.4%) | 32056 (40.7%) |
| >=30.0 | 2954 (21.9%) | 14859 (18.9%) |
| Missing | 127 (0.9%) | 565 (0.7%) |
| Ethnicity, n (%) |  |  |
| white | 12714 (94.4%) | 76343 (97.0%) |
| other | 643 (4.8%) | 2159 (2.7%) |
| Missing | 115 (0.9%) | 199 (0.3%) |
| Education, n (%) |  |  |
| college or University degree | 4911 (36.5%) | 35198 (44.7%) |
| secondary school | 5184 (38.5%) | 28819 (36.6%) |
| primary school | 1604 (11.9%) | 6066 (7.7%) |
| professional qualification | 1433 (10.6%) | 8259 (10.5%) |
| Missing | 340 (2.5%) | 359 (0.5%) |
| Employment, n (%) |  |  |
| employed | 6937 (51.5%) | 42450 (53.9%) |
| retired | 5435 (40.3%) | 32025 (40.7%) |
| inactive | 993 (7.4%) | 4021 (5.1%) |
| Missing | 107 (0.8%) | 205 (0.3%) |
| Household income, £/year, n (%) |  |  |
| less than 18000 | 2200 (16.3%) | 10204 (13.0%) |
| 18000 to 30999 | 3225 (23.9%) | 17897 (22.7%) |
| 31000 to 51999 | 3146 (23.4%) | 20549 (26.1%) |
| 52000 to 100000 | 2152 (16.0%) | 17621 (22.4%) |
| greater than 100000 | 401 (3.0%) | 5374 (6.8%) |
| Missing | 2348 (17.4%) | 7056 (9.0%) |
| Townsend deprivation index, mean (SD) | -1.40 (3.00) | -1.79 (2.78) |
| Missing | 12 (0.1%) | 90 (0.1%) |
| Smoking status, n (%) |  |  |
| never | 7549 (56.0%) | 45225 (57.5%) |
| previous | 4792 (35.6%) | 28421 (36.1%) |
| current | 1002 (7.4%) | 4814 (6.1%) |
| Missing | 129 (1.0%) | 241 (0.3%) |
| Alcohol drinking frequency, n (%) |  |  |
| >= 3 times/week | 5680 (42.2%) | 38070 (48.4%) |
| < 3 times/week | 6740 (50.0%) | 35853 (45.6%) |
| never | 978 (7.3%) | 4658 (5.9%) |
| Missing | 74 (0.5%) | 120 (0.2%) |
| Sleep duration, n (%) |  |  |
| 7-8 h/day | 8906 (66.1%) | 56123 (71.3%) |
| < 7h/day | 3437 (25.5%) | 17177 (21.8%) |
| > 8h/day | 958 (7.1%) | 5180 (6.6%) |
| Missing | 171 (1.3%) | 221 (0.3%) |
| Healthy diet score, n (%) |  |  |
| 0-2 | 4255 (31.6%) | 25009 (31.8%) |
| 3-5 | 7026 (52.2%) | 45353 (57.6%) |
| >=6 | 411 (3.1%) | 2698 (3.4%) |
| Missing | 1780 (13.2%) | 5641 (7.2%) |
| Family history of diabetes, n (%) |  |  |
| No | 10057 (74.7%) | 60359 (76.7%) |
| Yes | 3116 (23.1%) | 17187 (21.8%) |
| Missing | 299 (2.2%) | 1155 (1.5%) |
| Family history of CVD, n (%) |  |  |
| No | 2764 (20.5%) | 17143 (21.8%) |
| Yes | 10421 (77.4%) | 60418 (76.8%) |
| Missing | 287 (2.1%) | 1140 (1.4%) |
| Family history of cancer, n (%) |  |  |
| No | 8235 (61.1%) | 48614 (61.8%) |
| Yes | 4938 (36.7%) | 28932 (36.8%) |
| Missing | 299 (2.2%) | 1155 (1.5%) |
| Total wear days, mean (SD) | 6.72 (0.71) | 6.72 (0.71) |
| Seasonality, n (%) |  |  |
| Autumn | 4149 (30.8%) | 23334 (29.6%) |
| Spring | 2986 (22.2%) | 17907 (22.8%) |
| Summer | 3527 (26.2%) | 20788 (26.4%) |
| Winter | 2810 (20.9%) | 16672 (21.2%) |
| TVPA, mg, mean (SD) | 37.6 (11.7) | 38.4 (11.9) |
| MVPA, hour/day, mean (SD) | 0.474 (0.424) | 0.517 (0.447) |
| LPA, hour/day, mean (SD) | 5.38 (1.62) | 5.27 (1.58) |
| ST, hour/day, mean (SD) | 7.83 (1.60) | 7.98 (1.60) |

CVD, cardiovascular disease; TVPA, total volume of physical activity; MVPA, moderate- to- vigorous- intensity physical activity; LPA, light- intensity physical activity; ST, sedentary time; mg, milligravity; Health diet score was calculated based on self- reported servings of fruits, vegetables, whole grains, vegetable oil, fish, dairy, refined grains, unprocessed meats, processed meats and sugar- sweetened beverages. More details can be found in Additional file 1: Table S4.

Townsend Index (including measures of unemployment, non- car ownership, non- home ownership and household overcrowding), derived from respondents’ postcode was used as an indicator of area- level SES.

Employment status is categorized as employed (includes paid employment or self- employed, paid or voluntary work or student), retired, and inactive (includes looking after home and/or family, unable to work and unemployed).

Education is categorized as college or University degree, secondary school (includes A levels/AS levels or equivalent, O levels/GCSEs or equivalent, CSEs or equivalent), primary school, and professional qualification (NVQ or HND or HNC or equivalent, other professional qualifications).

**Table S2. Items used for constructing frailty index**

|  | **Item** | **Trait** | **coding** |
| --- | --- | --- | --- |
| Sensory | 1 | Glaucoma | yes=1, no=0, do not know/prefer not to answer=missing |
|  | 2 | Cataracts | yes=1, no=0, do not know/prefer not to answer=missing |
|  | 3 | Hearing difficulty | yes=1, completely deaf=1, no=0, do not know/prefer not to answer=missing |
| Cranial | 4 | Migraine | yes=1, no=0, do not know/prefer not to answer=missing |
|  | 5 | Dental problems | yes=1, no=0, do not know/prefer not to answer=missing |
| Mental wellbeing | 6 | Self-rated health | excellent=0, good=0.25, fair=0.5, poor=1, do not know/prefer not to answer=missing |
|  | 7 | Fatigue: frequency of tiredness / lethargy in last two weeks | not at all=0, several days=0.25, more than half the days=0.5, nearly every day=1, do not know/prefer not to answer=missing |
|  | 8 | Sleep: experience of sleeplessness/insomnia | never/rarely=0, sometimes=0.5, usually=1, prefer not to answer=missing |
|  | 9 | Depressed feelings: frequency in last two weeks | not at all=0, several days=0.5, more than half the days=0.75, nearly every day=1, do not know/prefer not to answer=missing |
|  | 10 | Self-described nervous personality | yes=1, no=0, do not know/prefer not to answer=missing |
|  | 11 | Severe anxiety/ panic attacks | yes=1, no=0, do not know/prefer not to answer=missing |
|  | 12 | Common to feel loneliness | yes=1, no=0, do not know/prefer not to answer=missing |
|  | 13 | Sense of misery (ever/never) | yes=1, no=0, do not know/prefer not to answer=missing |
| Infirmity | 14 | Infirmity: long-standing illness or disability | yes=1, no=0, do not know/prefer not to answer=missing |
|  | 15 | Falls in last year | no falls=0, only one fall=0.5, more than one fall=1, prefer not to answer=missing |
|  | 16 | Fractures/broken bones in last five years | yes=1, no=0, do not know/prefer not to answer=missing |
| Cardiometabolic | 17 | Diabetes | yes=1, no=0, do not know/prefer not to answer=missing |
|  | 18 | Myocardial infarction | yes=1, no=0, do not know/prefer not to answer=missing |
|  | 19 | Angina | yes=1, no=0, do not know/prefer not to answer=missing |
|  | 20 | Stroke | yes=1, no=0, do not know/prefer not to answer=missing |
|  | 21 | High blood pressure | yes=1, no=0, do not know/prefer not to answer=missing |
|  | 22 | Hypothyroidism | yes=1, no=0, do not know/prefer not to answer=missing |
|  | 23 | Deep-vein thrombosis | yes=1, no=0, do not know/prefer not to answer=missing |
|  | 24 | High cholesterol | yes=1, no=0, do not know/prefer not to answer=missing |
| Respiratory | 25 | Breathing: wheeze in last year | yes=1, no=0, do not know/prefer not to answer=missing |
|  | 26 | Pneumonia | yes=1, no=0, do not know/prefer not to answer=missing |
|  | 27 | Chronic bronchitis/emphysema | yes=1, no=0, do not know/prefer not to answer=missing |
|  | 28 | Asthma | yes=1, no=0, do not know/prefer not to answer=missing |
| Musculoskeletal | 29 | Rheumatoid arthritis | yes=1, no=0, do not know/prefer not to answer=missing |
|  | 30 | Osteoarthritis | yes=1, no=0, do not know/prefer not to answer=missing |
|  | 31 | Gout | yes=1, no=0, do not know/prefer not to answer=missing |
|  | 32 | Osteoporosis | yes=1, no=0, do not know/prefer not to answer=missing |
| Immunological | 33 | Hayfever, allergic rhinitis or eczema | yes=1, no=0, do not know/prefer not to answer=missing |
|  | 34 | Psoriasis | yes=1, no=0, do not know/prefer not to answer=missing |
| Cancer | 35 | Any cancer diagnosis | yes=1, no=0, do not know/prefer not to answer=missing |
|  | 36 | Multiple cancers diagnosed (number reported) | no cancer=0, one cancer only=0, more than one cancer=1, prefer not to answer=missing |
| Pain | 37 | Chest pain | yes=1, no=0, do not know/prefer not to answer=missing |
|  | 38 | Head and/or neck pain | yes=1, no=0, prefer not to answer=missing |
|  | 39 | Back pain | yes=1, no=0, prefer not to answer=missing |
|  | 40 | Stomach/abdominal pain | yes=1, no=0, prefer not to answer=missing |
|  | 41 | Hip pain | yes=1, no=0, prefer not to answer=missing |
|  | 42 | Knee pain | yes=1, no=0, prefer not to answer=missing |
|  | 43 | Whole-body pain | yes=1, no=0, prefer not to answer=missing |
|  | 44 | Facial pain | yes=1, no=0, prefer not to answer=missing |
|  | 45 | Sciatica | yes=1, no=0, do not know/prefer not to answer=missing |
| Gastrointestinal | 46 | Gastric reflux | yes=1, no=0, do not know/prefer not to answer=missing |
|  | 47 | Hiatus hernia | yes=1, no=0, do not know/prefer not to answer=missing |
|  | 48 | Gall stones | yes=1, no=0, do not know/prefer not to answer=missing |
|  | 49 | Diverticulitis | yes=1, no=0, do not know/prefer not to answer=missing |

**Table S3. The resource and definition of the covariates**

|  | **Field ID** | **Description** | **Coding** |
| --- | --- | --- | --- |
| Age at accelerometer measurement | 34,52,90010 | Age at accelerometer measurement was calculated from the date of birth (year 34, month 52) and the date of wearing the accelerometer (90010). | continuous |
| Sex | 31 | The existing variable 'sex' (31) was applied. | female, male |
| Body mass index | 21001 | The existing variable 'body mass index' (21001) was applied. | < 25.0, 25.0 – 29.9, ≥ 30.0 kg/m^2^ |
| Ethnicity | 21000 | The existing variable 'ethnic background' (21000) was applied. | white, others |
| Assessment center | 54 | The existing variable 'assessment center' (54) was applied. | England, Wales, Scotland |
| Education | 6138 | The existing variable 'qualifications' (6138) was applied. If one selected "college or university degree," they were classified as such. If not, and they chose "A levels/AS levels or equivalent/O levels/GCSEs or equivalent/CSEs or equivalent", they were labeled "secondary school." If neither applied but picked "NVQ or HND or HNC or equivalent/Other professional qualifications eg: nursing, teaching", they fell under "professional qualification." If none of these options but selected "None of the above," they were categorized as "primary school." For "NA" or "prefer not to answer," the designation was "NA." | college or university degree, secondary school, primary school, professional qualification |
| Employment | 6142, 20119 | We defined employment based on 'Current employment status' (6142), and 'Current employment status-corrected' (20119). If selected "retired," they were categorized as "retired." If not, and they chose "in paid employment or self-employed/Doing unpaid or voluntary work", they were classified as "employed." If none of the prior options were chosen but one of "Looking after home and/or family, Unable to work because of sickness or disability, Unemployed, Full or part-time student, None of the above" was selected, they were categorized as "inactive." For "NA" or "prefer not to answer," the designation was "NA." | employed, retired, inactive |
| Household income | 738 | The existing variable 'Average total household income before tax' (738) was applied. | < 18,000, 18,000–30,999, 31,000–51,999, 52,000–100,000, > £100,000 £/year |
| Townsend deprivation index | 189 | The existing variable 'Townsend deprivation index at recruitment' (189) was applied. | continuous |
| Smoking status | 20116 | The existing variable 'smoking status' (20116) was applied. | current, former, never |
| Alcohol drinking frequency | 1558 | The existing variable 'alcohol intake frequency' (1558) was applied. | >= 3 times/week, <3 times/week, never |
| Sleep duration | 1160 | The existing variable 'sleep duration' (1160) was applied. | <7h per day,7–8h per day, > 8h per day |
| Healthy diet score^*^ | - | - | - |
| Family history of diabetes | 20107, 20110, 20111 | We defined family history of diabetes based on 'Illnesses of mother' (20110), 'Illnesses of father' (20107). and 'Illnesses of siblings' (20111). | yes, no |
| Family history of CVD | 20107, 20110, 20111 | We defined family history of CVD based on 'Illnesses of mother' (20110), 'Illnesses of father' (20107). and 'Illnesses of siblings' (20111). | yes, no |
| Family history of cancer | 20107, 20110, 20111 | We defined family history of cancer based on 'Illnesses of mother' (20110), 'Illnesses of father' (20107). and 'Illnesses of siblings' (20111). | yes, no |

CVD: cardiovascular disease.

* Detailed information of constructing healthy diet score was presented in Additional file 1: Table S4. For further information, please refer to the UK Biobank data showcase https://biobank.ndph.ox.ac.uk/showcase/

**Table S4. Definition of each component of a healthy diet score**

| **Components** | **Goal (1 point)** | **Amount per serving** | **Field IDs** |
| --- | --- | --- | --- |
| Fruits | ≥ 3 servings/day | 1 piece of fresh fruit  5 pieces of dried fruit | 1309, 1319 |
| Vegetables | ≥ 3 servings/day | 3 heaped tablespoons | 1289, 1299 |
| Whole grains | ≥ 3 servings/day | 1 slice of whole-grain bread  1 cup of whole-grain cereal | 1438, 1448, 1458, 1468 |
| Vegetable oil | ≥ 2 servings/day | 1 serving/day if in combination with eating at least 2 slices of bread (ID 1438) | 1428 (Flora Pro-Active/Benecol spread), 2654 (Flora Pro-Active/Benecol, soft margarine -,olive oil based -, polyunsaturated/sunflower oil based -, other low/reduced fat spread), 1438 (bread slices/week) |
| (Shell)Fish | ≥ 2 servings/week | Once/week | 1329, 1339 |
| Dairy | ≥ 2 servings/day | 1 glass/day if consumption any type of milk  1 piece of cheese | 1408, 1418 |
| Refined grains | ≤ 2 servings/day | 1 slice of bread  1 bowl of cereal | 1438, 1448 (white, brown, other bread slices/week)  1458, 1468 (biscuit, other cereals/week) |
| Unprocessed meats | ≤ 2 servings/week | Once/week (including poultry, beef, lamb, and pork)  0 pieces/day if indicated having never eaten meat | 1359, 1369, 1379, 1389, 3680 |
| Processed meats | ≤ 1 servings/week | 1 piece/day  0 pieces/day if indicated having never eaten meat | 1349, 3680 |
| Sugar-sweetened beverages | Don’t drink | 0 serving | 6144 |

Data on food consumption were derived from the baseline questionnaire. If participants achieved the intake goal of each diet component, they were considered to have an adequate intake and get one point. The points were then accumulated to calculate the final healthy diet score. A higher score indicated a healthier diet pattern.

**Table S5. The numbers (percentages) of participants with missing covariates**

| **Covariates** | **N** | **%** |
| --- | --- | --- |
| Any missing covariates | 13737 | 17.50% |
| Age at accelerometer measurement | 0 | 0 |
| Sex | 0 | 0 |
| Body mass index (BMI) | 564 | 0.72% |
| Ethnicity | 199 | 0.25% |
| Education | 359 | 0.46% |
| Employment | 205 | 0.26% |
| Household income | 7047 | 8.98% |
| Townsend deprivation index | 90 | 0.11% |
| Assessment center | 0 | 0 |
| Smoking status | 241 | 0.31% |
| Alcohol drinking frequency | 120 | 0.15% |
| Sleep duration | 220 | 0.28% |
| Healthy diet score | 5622 | 7.16% |
| Family history of diabetes | 1150 | 1.46% |
| Family history of CVD | 1135 | 1.45% |
| Family history of cancer | 1150 | 1.46% |
| Seasonality | 0 | 0 |
| Total wear days | 0 | 0 |

**Table S6. Baseline characteristics of the participants stratified by frailty index (before imputation)**

|  | **Overall** | **Robust** | **Pre-frailty** | **Frailty** |
| --- | --- | --- | --- | --- |
|  | (N=78508) | (N=49736) | (N=25579) | (N=3193) |
| Age at accelerometer measurement, mean (SD) | 62.0 (7.8) | 61.5 (7.8) | 62.8 (7.7) | 63.5 (7.4) |
| Sex, n (%) |  |  |  |  |
| female | 43538 (55.5%) | 26757 (53.8%) | 14844 (58.0%) | 1937 (60.7%) |
| male | 34970 (44.5%) | 22979 (46.2%) | 10735 (42.0%) | 1256 (39.3%) |
| Body mass index (BMI), kg/m2, n (%) |  |  |  |  |
| <25.0 | 31116 (39.6%) | 22000 (44.2%) | 8523 (33.3%) | 593 (18.6%) |
| 25.0-29.9 | 31998 (40.8%) | 20318 (40.9%) | 10554 (41.3%) | 1126 (35.3%) |
| >=30.0 | 14830 (18.9%) | 7050 (14.2%) | 6334 (24.8%) | 1446 (45.3%) |
| Missing | 564 (0.7%) | 368 (0.7%) | 168 (0.7%) | 28 (0.9%) |
| Ethnicity, n (%) |  |  |  |  |
| white | 76170 (97.0%) | 48301 (97.1%) | 24799 (97.0%) | 3070 (96.1%) |
| other | 2139 (2.7%) | 1323 (2.7%) | 709 (2.8%) | 107 (3.4%) |
| Missing | 199 (0.3%) | 112 (0.2%) | 71 (0.3%) | 16 (0.5%) |
| Education, n (%) |  |  |  |  |
| college or University degree | 35109 (44.7%) | 23914 (48.1%) | 10254 (40.1%) | 941 (29.5%) |
| secondary school | 28727 (36.6%) | 17737 (35.7%) | 9721 (38.0%) | 1269 (39.7%) |
| primary school | 6064 (7.7%) | 2994 (6.0%) | 2548 (10.0%) | 522 (16.3%) |
| professional qualification | 8249 (10.5%) | 4881 (9.8%) | 2925 (11.4%) | 443 (13.9%) |
| Missing | 359 (0.5%) | 210 (0.4%) | 131 (0.5%) | 18 (0.6%) |
| Employment, n (%) |  |  |  |  |
| employed | 42277 (53.9%) | 28395 (57.1%) | 12705 (49.7%) | 1177 (36.9%) |
| retired | 32023 (40.8%) | 19306 (38.8%) | 11284 (44.1%) | 1433 (44.9%) |
| inactive | 4003 (5.1%) | 1916 (3.9%) | 1511 (5.9%) | 576 (18.0%) |
| Missing | 205 (0.3%) | 119 (0.2%) | 79 (0.3%) | 7 (0.2%) |
| Household income, £/year, n (%) |  |  |  |  |
| less than 18000 | 10189 (13.0%) | 4928 (9.9%) | 4295 (16.8%) | 966 (30.3%) |
| 18000 to 30999 | 17864 (22.8%) | 10657 (21.4%) | 6370 (24.9%) | 837 (26.2%) |
| 31000 to 51999 | 20502 (26.1%) | 13476 (27.1%) | 6414 (25.1%) | 612 (19.2%) |
| 52000 to 100000 | 17558 (22.4%) | 12364 (24.9%) | 4830 (18.9%) | 364 (11.4%) |
| greater than 100000 | 5348 (6.8%) | 4093 (8.2%) | 1194 (4.7%) | 61 (1.9%) |
| Missing | 7047 (9.0%) | 4218 (8.5%) | 2476 (9.7%) | 353 (11.1%) |
| Townsend deprivation index, mean (SD) | -1.79 (2.78) | -1.92 (2.70) | -1.64 (2.85) | -0.843 (3.21) |
| Missing | 90 (0.1%) | 49 (0.1%) | 39 (0.2%) | 2 (0.1%) |
| Smoking status, n (%) |  |  |  |  |
| never | 45104 (57.5%) | 30267 (60.9%) | 13388 (52.3%) | 1449 (45.4%) |
| previous | 28364 (36.1%) | 16708 (33.6%) | 10267 (40.1%) | 1389 (43.5%) |
| current | 4799 (6.1%) | 2623 (5.3%) | 1832 (7.2%) | 344 (10.8%) |
| Missing | 241 (0.3%) | 138 (0.3%) | 92 (0.4%) | 11 (0.3%) |
| Alcohol drinking frequency, n (%) |  |  |  |  |
| >= 3 times/week | 38006 (48.4%) | 25314 (50.9%) | 11653 (45.6%) | 1039 (32.5%) |
| < 3 times/week | 35736 (45.5%) | 21927 (44.1%) | 12066 (47.2%) | 1743 (54.6%) |
| never | 4646 (5.9%) | 2421 (4.9%) | 1819 (7.1%) | 406 (12.7%) |
| Missing | 120 (0.2%) | 74 (0.1%) | 41 (0.2%) | 5 (0.2%) |
| Sleep duration, n (%) |  |  |  |  |
| 7-8 h/day | 55982 (71.3%) | 37649 (75.7%) | 16767 (65.5%) | 1566 (49.0%) |
| < 7h/day | 17138 (21.8%) | 9210 (18.5%) | 6734 (26.3%) | 1194 (37.4%) |
| > 8h/day | 5168 (6.6%) | 2769 (5.6%) | 1991 (7.8%) | 408 (12.8%) |
| Missing | 220 (0.3%) | 108 (0.2%) | 87 (0.3%) | 25 (0.8%) |
| Healthy diet score, n (%) |  |  |  |  |
| 0-2 | 24930 (31.8%) | 15394 (31.0%) | 8446 (33.0%) | 1090 (34.1%) |
| 3-5 | 45263 (57.7%) | 29272 (58.9%) | 14315 (56.0%) | 1676 (52.5%) |
| >=6 | 2693 (3.4%) | 1826 (3.7%) | 772 (3.0%) | 95 (3.0%) |
| Missing | 5622 (7.2%) | 3244 (6.5%) | 2046 (8.0%) | 332 (10.4%) |
| Family history of diabetes, n (%) |  |  |  |  |
| No | 60218 (76.7%) | 38975 (78.4%) | 19061 (74.5%) | 2182 (68.3%) |
| Yes | 17140 (21.8%) | 10129 (20.4%) | 6077 (23.8%) | 934 (29.3%) |
| Missing | 1150 (1.5%) | 632 (1.3%) | 441 (1.7%) | 77 (2.4%) |
| Family history of CVD, n (%) |  |  |  |  |
| No | 17087 (21.8%) | 11881 (23.9%) | 4783 (18.7%) | 423 (13.2%) |
| Yes | 60286 (76.8%) | 37231 (74.9%) | 20361 (79.6%) | 2694 (84.4%) |
| Missing | 1135 (1.4%) | 624 (1.3%) | 435 (1.7%) | 76 (2.4%) |
| Family history of cancer, n (%) |  |  |  |  |
| No | 48475 (61.7%) | 31153 (62.6%) | 15413 (60.3%) | 1909 (59.8%) |
| Yes | 28883 (36.8%) | 17952 (36.1%) | 9724 (38.0%) | 1207 (37.8%) |
| Missing | 1150 (1.5%) | 631 (1.3%) | 442 (1.7%) | 77 (2.4%) |
| Total wear days, mean (SD) | 6.72(0.71) | 6.73(0.71) | 6.72(0.72) | 6.73(0.69) |
| Seasonality, n (%) |  |  |  |  |
| Autumn | 23285 (29.7%) | 14854 (29.9%) | 7472 (29.2%) | 959 (30.0%) |
| Spring | 17873 (22.8%) | 11361 (22.8%) | 5785 (22.6%) | 727 (22.8%) |
| Summer | 20730 (26.4%) | 12930 (26.0%) | 6972 (27.3%) | 828 (25.9%) |
| Winter | 16620 (21.2%) | 10591 (21.3%) | 5350 (20.9%) | 679 (21.3%) |
| TVPA, mg, mean (SD) | 38.4 (11.9) | 39.7 (11.9) | 36.5 (11.3) | 32.0 (10.6) |
| MVPA, hour/day, mean (SD) | 0.517 (0.447) | 0.568 (0.462) | 0.445 (0.410) | 0.297 (0.340) |
| LPA, hour/day, mean (SD) | 5.27 (1.58) | 5.32 (1.56) | 5.22 (1.59) | 4.85 (1.70) |
| ST, hour/day, mean (SD) | 7.98 (1.60) | 7.97 (1.59) | 7.98 (1.60) | 8.07 (1.69) |

CVD, cardiovascular disease; TVPA, total volume of physical activity; MVPA, moderate- to- vigorous- intensity physical activity; LPA, light- intensity physical activity; ST, sedentary time; mg, milligravity; Health diet score was calculated based on self- reported servings of fruits, vegetables, whole grains, vegetable oil, fish, dairy, refined grains, unprocessed meats, processed meats and sugar- sweetened beverages. More details can be found in Additional file 1: Table S4.

Townsend Index (including measures of unemployment, non- car ownership, non- home ownership and household overcrowding), derived from respondents’ postcode was used as an indicator of area- level SES.

Employment status is categorized as employed (includes paid employment or self- employed, paid or voluntary work or student), retired, and inactive (includes looking after home and/or family, unable to work and unemployed).

Education is categorized as college or University degree, secondary school (includes A levels/AS levels or equivalent, O levels/GCSEs or equivalent, CSEs or equivalent), primary school, and professional qualification (NVQ or HND or HNC or equivalent, other professional qualifications).

**Table S7. Baseline characteristics of the participants stratified by TVPA**

|  | **Overall** | **Low tertile** | **Medium tertile** | **Top tertile** |
| --- | --- | --- | --- | --- |
|  | (N=78508) | (N=26170) | (N=26169) | (N=26169) |
| Age at accelerometer measurement, mean (SD) | 62.0 (7.8) | 64.2 (7.4) | 62.0 (7.7) | 59.6 (7.7) |
| Sex |  |  |  |  |
| female | 43538 (55.5%) | 13015 (49.7%) | 15090 (57.7%) | 15433 (59.0%) |
| male | 34970 (44.5%) | 13155 (50.3%) | 11079 (42.3%) | 10736 (41.0%) |
| Body mass index (BMI), kg/m^2^, n (%) |  |  |  |  |
| <25.0 | 31356 (39.9%) | 7262 (27.7%) | 10335 (39.5%) | 13759 (52.6%) |
| 25.0-29.9 | 32136 (40.9%) | 11179 (42.7%) | 11290 (43.1%) | 9667 (36.9%) |
| >=30.0 | 15016 (19.1%) | 7729 (29.5%) | 4544 (17.4%) | 2743 (10.5%) |
| Ethnicity, n (%) |  |  |  |  |
| white | 76355 (97.3%) | 25549 (97.6%) | 25439 (97.2%) | 25367 (96.9%) |
| other | 2153 (2.7%) | 621 (2.4%) | 730 (2.8%) | 802 (3.1%) |
| Education, n (%) |  |  |  |  |
| college or University degree | 35182 (44.8%) | 11258 (43.0%) | 11870 (45.4%) | 12054 (46.1%) |
| secondary school | 28803 (36.7%) | 9259 (35.4%) | 9644 (36.9%) | 9900 (37.8%) |
| primary school | 6203 (7.9%) | 2569 (9.8%) | 1940 (7.4%) | 1694 (6.5%) |
| professional qualification | 8320 (10.6%) | 3084 (11.8%) | 2715 (10.4%) | 2521 (9.6%) |
| Employment, n (%) |  |  |  |  |
| employed | 42369 (54.0%) | 11639 (44.5%) | 14274 (54.5%) | 16456 (62.9%) |
| retired | 32111 (40.9%) | 13121 (50.1%) | 10730 (41.0%) | 8260 (31.6%) |
| inactive | 4028 (5.1%) | 1410 (5.4%) | 1165 (4.5%) | 1453 (5.6%) |
| Household income, £/year, n (%) |  |  |  |  |
| less than 18000 | 12472 (15.9%) | 5280 (20.2%) | 3880 (14.8%) | 3312 (12.7%) |
| 18000 to 30999 | 19679 (25.1%) | 7264 (27.8%) | 6515 (24.9%) | 5900 (22.5%) |
| 31000 to 51999 | 21841 (27.8%) | 6845 (26.2%) | 7415 (28.3%) | 7581 (29.0%) |
| 52000 to 100000 | 18315 (23.3%) | 5208 (19.9%) | 6229 (23.8%) | 6878 (26.3%) |
| greater than 100000 | 6201 (7.9%) | 1573 (6.0%) | 2130 (8.1%) | 2498 (9.5%) |
| Townsend deprivation index, mean (SD) | -1.79 (2.78) | -1.64 (2.87) | -1.86 (2.74) | -1.86 (2.71) |
| Smoking status, n (%) |  |  |  |  |
| never | 45193 (57.6%) | 14075 (53.8%) | 15315 (58.5%) | 15803 (60.4%) |
| previous | 28468 (36.3%) | 9988 (38.2%) | 9412 (36.0%) | 9068 (34.7%) |
| current | 4847 (6.2%) | 2107 (8.1%) | 1442 (5.5%) | 1298 (5.0%) |
| Alcohol drinking frequency, n (%) |  |  |  |  |
| >= 3 times/week | 38041 (48.5%) | 11892 (45.4%) | 12954 (49.5%) | 13195 (50.4%) |
| < 3 times/week | 35795 (45.6%) | 12459 (47.6%) | 11779 (45.0%) | 11557 (44.2%) |
| never | 4672 (6.0%) | 1819 (7.0%) | 1436 (5.5%) | 1417 (5.4%) |
| Sleep duration, n (%) |  |  |  |  |
| 7-8 h/day | 56048 (71.4%) | 17861 (68.3%) | 18813 (71.9%) | 19374 (74.0%) |
| < 7h/day | 17197 (21.9%) | 5911 (22.6%) | 5685 (21.7%) | 5601 (21.4%) |
| > 8h/day | 5263 (6.7%) | 2398 (9.2%) | 1671 (6.4%) | 1194 (4.6%) |
| Healthy diet score, n (%) |  |  |  |  |
| 0-2 | 27185 (34.6%) | 9836 (37.6%) | 8945 (34.2%) | 8404 (32.1%) |
| 3-5 | 47762 (60.8%) | 15356 (58.7%) | 16043 (61.3%) | 16363 (62.5%) |
| >=6 | 3561 (4.5%) | 978 (3.7%) | 1181 (4.5%) | 1402 (5.4%) |
| Family history of diabetes, n (%) |  |  |  |  |
| No | 60987 (77.7%) | 20099 (76.8%) | 20308 (77.6%) | 20580 (78.6%) |
| Yes | 17521 (22.3%) | 6071 (23.2%) | 5861 (22.4%) | 5589 (21.4%) |
| Family history of CVD, n (%) |  |  |  |  |
| No | 17621 (22.4%) | 5625 (21.5%) | 5750 (22.0%) | 6246 (23.9%) |
| Yes | 60887 (77.6%) | 20545 (78.5%) | 20419 (78.0%) | 19923 (76.1%) |
| Family history of cancer, n (%) |  |  |  |  |
| No | 49063 (62.5%) | 16126 (61.6%) | 16349 (62.5%) | 16588 (63.4%) |
| Yes | 29445 (37.5%) | 10044 (38.4%) | 9820 (37.5%) | 9581 (36.6%) |
| Total wear days, mean (SD) | 6.72 (0.709) | 6.73 (0.703) | 6.73 (0.702) | 6.71 (0.723) |
| Seasonality, n (%) |  |  |  |  |
| Autumn | 23285 (29.7%) | 7919 (30.3%) | 7773 (29.7%) | 7593 (29.0%) |
| Spring | 17873 (22.8%) | 5522 (21.1%) | 5920 (22.6%) | 6431 (24.6%) |
| Summer | 20730 (26.4%) | 6348 (24.3%) | 6961 (26.6%) | 7421 (28.4%) |
| Winter | 16620 (21.2%) | 6381 (24.4%) | 5515 (21.1%) | 4724 (18.1%) |
| MVPA, hour/day, mean (SD) | 0.517 (0.447) | 0.297 (0.263) | 0.486 (0.357) | 0.768 (0.540) |
| LPA, hour/day, mean (SD) | 5.27 (1.58) | 4.10 (1.15) | 5.35 (1.18) | 6.36 (1.49) |
| ST, hour/day, mean (SD) | 7.98 (1.60) | 8.87 (1.45) | 8.01 (1.34) | 7.06 (1.45) |

CVD, cardiovascular disease; TVPA, total volume of physical activity; MVPA, moderate- to- vigorous- intensity physical activity; LPA, light- intensity physical activity; ST, sedentary time; mg, milligravity; Health diet score was calculated based on self- reported servings of fruits, vegetables, whole grains, vegetable oil, fish, dairy, refined grains, unprocessed meats, processed meats and sugar- sweetened beverages. More details can be found in Additional file 1: Table S4.

Townsend Index (including measures of unemployment, non- car ownership, non- home ownership and household overcrowding), derived from respondents’ postcode was used as an indicator of area- level SES.

Employment status is categorized as employed (includes paid employment or self- employed, paid or voluntary work or student), retired, and inactive (includes looking after home and/or family, unable to work and unemployed).

Education is categorized as college or University degree, secondary school (includes A levels/AS levels or equivalent, O levels/GCSEs or equivalent, CSEs or equivalent), primary school, and professional qualification (NVQ or HND or HNC or equivalent, other professional qualifications).

**Table S8. Baseline characteristics of the participants stratified by MVPA**

|  | **Overall** | **Low tertile** | **Medium tertile** | **Top tertile** |
| --- | --- | --- | --- | --- |
|  | (N=78508) | (N=26232) | (N=26140) | (N=26136) |
| Age at accelerometer measurement, mean (SD) | 62.0 (7.8) | 63.1 (7.7) | 61.9 (7.8) | 60.9 (7.7) |
| Sex |  |  |  |  |
| female | 43538 (55.5%) | 16852 (64.2%) | 14842 (56.8%) | 11844 (45.3%) |
| male | 34970 (44.5%) | 9380 (35.8%) | 11298 (43.2%) | 14292 (54.7%) |
| Body mass index (BMI), kg/m^2^, n (%) |  |  |  |  |
| <25.0 | 31356 (39.9%) | 7771 (29.6%) | 10722 (41.0%) | 12863 (49.2%) |
| 25.0-29.9 | 32136 (40.9%) | 10752 (41.0%) | 10964 (41.9%) | 10420 (39.9%) |
| >=30.0 | 15016 (19.1%) | 7709 (29.4%) | 4454 (17.0%) | 2853 (10.9%) |
| Ethnicity, n (%) |  |  |  |  |
| white | 76355 (97.3%) | 25449 (97.0%) | 25400 (97.2%) | 25506 (97.6%) |
| other | 2153 (2.7%) | 783 (3.0%) | 740 (2.8%) | 630 (2.4%) |
| Education, n (%) |  |  |  |  |
| college or University degree | 35182 (44.8%) | 9235 (35.2%) | 11788 (45.1%) | 14159 (54.2%) |
| secondary school | 28803 (36.7%) | 10933 (41.7%) | 9735 (37.2%) | 8135 (31.1%) |
| primary school | 6203 (7.9%) | 2894 (11.0%) | 1918 (7.3%) | 1391 (5.3%) |
| professional qualification | 8320 (10.6%) | 3170 (12.1%) | 2699 (10.3%) | 2451 (9.4%) |
| Employment, n (%) |  |  |  |  |
| employed | 42369 (54.0%) | 12803 (48.8%) | 14371 (55.0%) | 15195 (58.1%) |
| retired | 32111 (40.9%) | 11834 (45.1%) | 10543 (40.3%) | 9734 (37.2%) |
| inactive | 4028 (5.1%) | 1595 (6.1%) | 1226 (4.7%) | 1207 (4.6%) |
| Household income, £/year, n (%) |  |  |  |  |
| less than 18000 | 12472 (15.9%) | 5409 (20.6%) | 3855 (14.7%) | 3208 (12.3%) |
| 18000 to 30999 | 19679 (25.1%) | 7431 (28.3%) | 6596 (25.2%) | 5652 (21.6%) |
| 31000 to 51999 | 21841 (27.8%) | 7097 (27.1%) | 7332 (28.0%) | 7412 (28.4%) |
| 52000 to 100000 | 18315 (23.3%) | 4954 (18.9%) | 6282 (24.0%) | 7079 (27.1%) |
| greater than 100000 | 6201 (7.9%) | 1341 (5.1%) | 2075 (7.9%) | 2785 (10.7%) |
| Townsend deprivation index, mean (SD) | -1.79 (2.78) | -1.88 (2.73) | -1.83 (2.75) | -1.65 (2.85) |
| Smoking status, n (%) |  |  |  |  |
| never | 45193 (57.6%) | 14224 (54.2%) | 15491 (59.3%) | 15478 (59.2%) |
| previous | 28468 (36.3%) | 9889 (37.7%) | 9168 (35.1%) | 9411 (36.0%) |
| current | 4847 (6.2%) | 2119 (8.1%) | 1481 (5.7%) | 1247 (4.8%) |
| Alcohol drinking frequency, n (%) |  |  |  |  |
| >= 3 times/week | 38041 (48.5%) | 11030 (42.0%) | 12773 (48.9%) | 14238 (54.5%) |
| < 3 times/week | 35795 (45.6%) | 13249 (50.5%) | 11930 (45.6%) | 10616 (40.6%) |
| never | 4672 (6.0%) | 1953 (7.4%) | 1437 (5.5%) | 1282 (4.9%) |
| Sleep duration, n (%) |  |  |  |  |
| 7-8 h/day | 56048 (71.4%) | 17684 (67.4%) | 18802 (71.9%) | 19562 (74.8%) |
| < 7h/day | 17197 (21.9%) | 6348 (24.2%) | 5652 (21.6%) | 5197 (19.9%) |
| > 8h/day | 5263 (6.7%) | 2200 (8.4%) | 1686 (6.4%) | 1377 (5.3%) |
| Healthy diet score, n (%) |  |  |  |  |
| 0-2 | 27185 (34.6%) | 9811 (37.4%) | 8993 (34.4%) | 8381 (32.1%) |
| 3-5 | 47762 (60.8%) | 15466 (59.0%) | 16004 (61.2%) | 16292 (62.3%) |
| >=6 | 3561 (4.5%) | 955 (3.6%) | 1143 (4.4%) | 1463 (5.6%) |
| Family history of diabetes, n (%) |  |  |  |  |
| No | 60987 (77.7%) | 19860 (75.7%) | 20353 (77.9%) | 20774 (79.5%) |
| Yes | 17521 (22.3%) | 6372 (24.3%) | 5787 (22.1%) | 5362 (20.5%) |
| Family history of CVD, n (%) |  |  |  |  |
| No | 17621 (22.4%) | 5538 (21.1%) | 5848 (22.4%) | 6235 (23.9%) |
| Yes | 60887 (77.6%) | 20694 (78.9%) | 20292 (77.6%) | 19901 (76.1%) |
| Family history of cancer, n (%) |  |  |  |  |
| No | 49063 (62.5%) | 16213 (61.8%) | 16424 (62.8%) | 16426 (62.8%) |
| Yes | 29445 (37.5%) | 10019 (38.2%) | 9716 (37.2%) | 9710 (37.2%) |
| Total wear days, mean (SD) | 6.72 (0.709) | 6.70 (0.735) | 6.74 (0.687) | 6.73 (0.704) |
| Seasonality, n (%) |  |  |  |  |
| Autumn | 23285 (29.7%) | 7914 (30.2%) | 7742 (29.6%) | 7629 (29.2%) |
| Spring | 17873 (22.8%) | 5529 (21.1%) | 5916 (22.6%) | 6428 (24.6%) |
| Summer | 20730 (26.4%) | 6381 (24.3%) | 6840 (26.2%) | 7509 (28.7%) |
| Winter | 16620 (21.2%) | 6408 (24.4%) | 5642 (21.6%) | 4570 (17.5%) |
| TVPA, mg, mean (SD) | 38.4 (11.9) | 32.4 (9.43) | 37.6 (9.66) | 45.1 (12.6) |
| LPA, hour/day, mean (SD) | 5.27 (1.58) | 5.17 (1.68) | 5.38 (1.57) | 5.27 (1.47) |
| ST, hour/day, mean (SD) | 7.98 (1.60) | 8.21 (1.64) | 8.00 (1.58) | 7.72 (1.52) |

CVD, cardiovascular disease; TVPA, total volume of physical activity; MVPA, moderate- to- vigorous- intensity physical activity; LPA, light- intensity physical activity; ST, sedentary time; mg, milligravity; Health diet score was calculated based on self- reported servings of fruits, vegetables, whole grains, vegetable oil, fish, dairy, refined grains, unprocessed meats, processed meats and sugar- sweetened beverages. More details can be found in Additional file 1: Table S4.

Townsend Index (including measures of unemployment, non- car ownership, non- home ownership and household overcrowding), derived from respondents’ postcode was used as an indicator of area- level SES.

Employment status is categorized as employed (includes paid employment or self- employed, paid or voluntary work or student), retired, and inactive (includes looking after home and/or family, unable to work and unemployed).

Education is categorized as college or University degree, secondary school (includes A levels/AS levels or equivalent, O levels/GCSEs or equivalent, CSEs or equivalent), primary school, and professional qualification (NVQ or HND or HNC or equivalent, other professional qualifications).

**Table S9. Baseline characteristics of the participants stratified by LPA**

|  | **Overall** | **Low tertile** | **Medium tertile** | **Top tertile** |
| --- | --- | --- | --- | --- |
|  | (N=78508) | (N=26184) | (N=26155) | (N=26169) |
| Age at accelerometer measurement, mean (SD) | 62.0 (7.8) | 61.6 (8.0) | 62.3 (7.7) | 62.0 (7.7) |
| Sex |  |  |  |  |
| female | 43538 (55.5%) | 10907 (41.7%) | 14963 (57.2%) | 17668 (67.5%) |
| male | 34970 (44.5%) | 15277 (58.3%) | 11192 (42.8%) | 8501 (32.5%) |
| Body mass index (BMI), kg/m^2^, n (%) |  |  |  |  |
| <25.0 | 31356 (39.9%) | 8490 (32.4%) | 10492 (40.1%) | 12374 (47.3%) |
| 25.0-29.9 | 32136 (40.9%) | 11074 (42.3%) | 10954 (41.9%) | 10108 (38.6%) |
| >=30.0 | 15016 (19.1%) | 6620 (25.3%) | 4709 (18.0%) | 3687 (14.1%) |
| Ethnicity, n (%) |  |  |  |  |
| white | 76355 (97.3%) | 25485 (97.3%) | 25481 (97.4%) | 25389 (97.0%) |
| other | 2153 (2.7%) | 699 (2.7%) | 674 (2.6%) | 780 (3.0%) |
| Education, n (%) |  |  |  |  |
| college or University degree | 35182 (44.8%) | 13011 (49.7%) | 11885 (45.4%) | 10286 (39.3%) |
| secondary school | 28803 (36.7%) | 8575 (32.7%) | 9521 (36.4%) | 10707 (40.9%) |
| primary school | 6203 (7.9%) | 1887 (7.2%) | 2005 (7.7%) | 2311 (8.8%) |
| professional qualification | 8320 (10.6%) | 2711 (10.4%) | 2744 (10.5%) | 2865 (10.9%) |
| Employment, n (%) |  |  |  |  |
| employed | 42369 (54.0%) | 14728 (56.2%) | 13786 (52.7%) | 13855 (52.9%) |
| retired | 32111 (40.9%) | 10117 (38.6%) | 11233 (42.9%) | 10761 (41.1%) |
| inactive | 4028 (5.1%) | 1339 (5.1%) | 1136 (4.3%) | 1553 (5.9%) |
| Household income, £/year, n (%) |  |  |  |  |
| less than 18000 | 12472 (15.9%) | 4080 (15.6%) | 4002 (15.3%) | 4390 (16.8%) |
| 18000 to 30999 | 19679 (25.1%) | 6052 (23.1%) | 6538 (25.0%) | 7089 (27.1%) |
| 31000 to 51999 | 21841 (27.8%) | 6939 (26.5%) | 7329 (28.0%) | 7573 (28.9%) |
| 52000 to 100000 | 18315 (23.3%) | 6633 (25.3%) | 6218 (23.8%) | 5464 (20.9%) |
| greater than 100000 | 6201 (7.9%) | 2480 (9.5%) | 2068 (7.9%) | 1653 (6.3%) |
| Townsend deprivation index, mean (SD) | -1.79 (2.78) | -1.53 (2.92) | -1.88 (2.73) | -1.94 (2.66) |
| Smoking status, n (%) |  |  |  |  |
| never | 45193 (57.6%) | 14723 (56.2%) | 15079 (57.7%) | 15391 (58.8%) |
| previous | 28468 (36.3%) | 9498 (36.3%) | 9609 (36.7%) | 9361 (35.8%) |
| current | 4847 (6.2%) | 1963 (7.5%) | 1467 (5.6%) | 1417 (5.4%) |
| Alcohol drinking frequency, n (%) |  |  |  |  |
| >= 3 times/week | 38041 (48.5%) | 12645 (48.3%) | 12924 (49.4%) | 12472 (47.7%) |
| < 3 times/week | 35795 (45.6%) | 11982 (45.8%) | 11744 (44.9%) | 12069 (46.1%) |
| never | 4672 (6.0%) | 1557 (5.9%) | 1487 (5.7%) | 1628 (6.2%) |
| Sleep duration, n (%) |  |  |  |  |
| 7-8 h/day | 56048 (71.4%) | 18362 (70.1%) | 18801 (71.9%) | 18885 (72.2%) |
| < 7h/day | 17197 (21.9%) | 5778 (22.1%) | 5581 (21.3%) | 5838 (22.3%) |
| > 8h/day | 5263 (6.7%) | 2044 (7.8%) | 1773 (6.8%) | 1446 (5.5%) |
| Healthy diet score, n (%) |  |  |  |  |
| 0-2 | 27185 (34.6%) | 10191 (38.9%) | 8742 (33.4%) | 8252 (31.5%) |
| 3-5 | 47762 (60.8%) | 15040 (57.4%) | 16204 (62.0%) | 16518 (63.1%) |
| >=6 | 3561 (4.5%) | 953 (3.6%) | 1209 (4.6%) | 1399 (5.3%) |
| Family history of diabetes, n (%) |  |  |  |  |
| No | 60987 (77.7%) | 20252 (77.3%) | 20273 (77.5%) | 20462 (78.2%) |
| Yes | 17521 (22.3%) | 5932 (22.7%) | 5882 (22.5%) | 5707 (21.8%) |
| Family history of CVD, n (%) |  |  |  |  |
| No | 17621 (22.4%) | 6082 (23.2%) | 5704 (21.8%) | 5835 (22.3%) |
| Yes | 60887 (77.6%) | 20102 (76.8%) | 20451 (78.2%) | 20334 (77.7%) |
| Family history of cancer, n (%) |  |  |  |  |
| No | 49063 (62.5%) | 16493 (63.0%) | 16249 (62.1%) | 16321 (62.4%) |
| Yes | 29445 (37.5%) | 9691 (37.0%) | 9906 (37.9%) | 9848 (37.6%) |
| Total wear days, mean (SD) | 6.72 (0.709) | 6.71 (0.724) | 6.73 (0.700) | 6.73 (0.704) |
| Seasonality, n (%) |  |  |  |  |
| Autumn | 23285 (29.7%) | 7856 (30.0%) | 7846 (30.0%) | 7583 (29.0%) |
| Spring | 17873 (22.8%) | 5745 (21.9%) | 5999 (22.9%) | 6129 (23.4%) |
| Summer | 20730 (26.4%) | 6578 (25.1%) | 6833 (26.1%) | 7319 (28.0%) |
| Winter | 16620 (21.2%) | 6005 (22.9%) | 5477 (20.9%) | 5138 (19.6%) |
| TVPA, mg, mean (SD) | 38.4 (11.9) | 30.8 (9.83) | 38.1 (9.37) | 46.1 (11.0) |
| MVPA, hour/day, mean (SD) | 0.517 (0.447) | 0.506 (0.466) | 0.539 (0.454) | 0.506 (0.420) |
| ST, hour/day, mean (SD) | 7.98 (1.60) | 9.29 (1.31) | 8.07 (1.00) | 6.58 (1.10) |

CVD, cardiovascular disease; TVPA, total volume of physical activity; MVPA, moderate- to- vigorous- intensity physical activity; LPA, light- intensity physical activity; ST, sedentary time; mg, milligravity; Health diet score was calculated based on self- reported servings of fruits, vegetables, whole grains, vegetable oil, fish, dairy, refined grains, unprocessed meats, processed meats and sugar- sweetened beverages. More details can be found in Additional file 1: Table S4.

Townsend Index (including measures of unemployment, non- car ownership, non- home ownership and household overcrowding), derived from respondents’ postcode was used as an indicator of area- level SES.

Employment status is categorized as employed (includes paid employment or self- employed, paid or voluntary work or student), retired, and inactive (includes looking after home and/or family, unable to work and unemployed).

Education is categorized as college or University degree, secondary school (includes A levels/AS levels or equivalent, O levels/GCSEs or equivalent, CSEs or equivalent), primary school, and professional qualification (NVQ or HND or HNC or equivalent, other professional qualifications).

**Table S10. Baseline characteristics of the participants stratified by ST**

|  | **Overall** | **Low tertile** | **Medium tertile** | **Top tertile** |
| --- | --- | --- | --- | --- |
|  | (N=78508) | (N=26170) | (N=26175) | (N=26163) |
| Age at accelerometer measurement, mean (SD) | 62.0 (7.8) | 62.3 (7.5) | 62.5 (7.7) | 61.1 (8.1) |
| Sex |  |  |  |  |
| female | 43538 (55.5%) | 16679 (63.7%) | 14932 (57.0%) | 11927 (45.6%) |
| male | 34970 (44.5%) | 9491 (36.3%) | 11243 (43.0%) | 14236 (54.4%) |
| Body mass index (BMI), kg/m^2^, n (%) |  |  |  |  |
| <25.0 | 31356 (39.9%) | 12079 (46.2%) | 10538 (40.3%) | 8739 (33.4%) |
| 25.0-29.9 | 32136 (40.9%) | 10251 (39.2%) | 10871 (41.5%) | 11014 (42.1%) |
| >=30.0 | 15016 (19.1%) | 3840 (14.7%) | 4766 (18.2%) | 6410 (24.5%) |
| Ethnicity, n (%) |  |  |  |  |
| white | 76355 (97.3%) | 25329 (96.8%) | 25524 (97.5%) | 25502 (97.5%) |
| other | 2153 (2.7%) | 841 (3.2%) | 651 (2.5%) | 661 (2.5%) |
| Education, n (%) |  |  |  |  |
| college or University degree | 35182 (44.8%) | 10264 (39.2%) | 11736 (44.8%) | 13182 (50.4%) |
| secondary school | 28803 (36.7%) | 10413 (39.8%) | 9531 (36.4%) | 8859 (33.9%) |
| primary school | 6203 (7.9%) | 2508 (9.6%) | 2103 (8.0%) | 1592 (6.1%) |
| professional qualification | 8320 (10.6%) | 2985 (11.4%) | 2805 (10.7%) | 2530 (9.7%) |
| Employment, n (%) |  |  |  |  |
| employed | 42369 (54.0%) | 13256 (50.7%) | 13370 (51.1%) | 15743 (60.2%) |
| retired | 32111 (40.9%) | 11166 (42.7%) | 11575 (44.2%) | 9370 (35.8%) |
| inactive | 4028 (5.1%) | 1748 (6.7%) | 1230 (4.7%) | 1050 (4.0%) |
| Household income, £/year, n (%) |  |  |  |  |
| less than 18000 | 12472 (15.9%) | 4884 (18.7%) | 4151 (15.9%) | 3437 (13.1%) |
| 18000 to 30999 | 19679 (25.1%) | 7252 (27.7%) | 6717 (25.7%) | 5710 (21.8%) |
| 31000 to 51999 | 21841 (27.8%) | 7354 (28.1%) | 7399 (28.3%) | 7088 (27.1%) |
| 52000 to 100000 | 18315 (23.3%) | 5152 (19.7%) | 5954 (22.7%) | 7209 (27.6%) |
| greater than 100000 | 6201 (7.9%) | 1528 (5.8%) | 1954 (7.5%) | 2719 (10.4%) |
| Townsend deprivation index, mean (SD) | -1.79 (2.78) | -1.82 (2.76) | -1.86 (2.74) | -1.69 (2.83) |
| Smoking status, n (%) |  |  |  |  |
| never | 45193 (57.6%) | 14927 (57.0%) | 15030 (57.4%) | 15236 (58.2%) |
| previous | 28468 (36.3%) | 9548 (36.5%) | 9622 (36.8%) | 9298 (35.5%) |
| current | 4847 (6.2%) | 1695 (6.5%) | 1523 (5.8%) | 1629 (6.2%) |
| Alcohol drinking frequency, n (%) |  |  |  |  |
| >= 3 times/week | 38041 (48.5%) | 12416 (47.4%) | 12921 (49.4%) | 12704 (48.6%) |
| < 3 times/week | 35795 (45.6%) | 12040 (46.0%) | 11773 (45.0%) | 11982 (45.8%) |
| never | 4672 (6.0%) | 1714 (6.5%) | 1481 (5.7%) | 1477 (5.6%) |
| Sleep duration, n (%) |  |  |  |  |
| 7-8 h/day | 56048 (71.4%) | 18726 (71.6%) | 18849 (72.0%) | 18473 (70.6%) |
| < 7h/day | 17197 (21.9%) | 5440 (20.8%) | 5550 (21.2%) | 6207 (23.7%) |
| > 8h/day | 5263 (6.7%) | 2004 (7.7%) | 1776 (6.8%) | 1483 (5.7%) |
| Healthy diet score, n (%) |  |  |  |  |
| 0-2 | 27185 (34.6%) | 8404 (32.1%) | 8769 (33.5%) | 10012 (38.3%) |
| 3-5 | 47762 (60.8%) | 16369 (62.5%) | 16168 (61.8%) | 15225 (58.2%) |
| >=6 | 3561 (4.5%) | 1397 (5.3%) | 1238 (4.7%) | 926 (3.5%) |
| Family history of diabetes, n (%) |  |  |  |  |
| No | 60987 (77.7%) | 20436 (78.1%) | 20369 (77.8%) | 20182 (77.1%) |
| Yes | 17521 (22.3%) | 5734 (21.9%) | 5806 (22.2%) | 5981 (22.9%) |
| Family history of CVD, n (%) |  |  |  |  |
| No | 17621 (22.4%) | 5814 (22.2%) | 5732 (21.9%) | 6075 (23.2%) |
| Yes | 60887 (77.6%) | 20356 (77.8%) | 20443 (78.1%) | 20088 (76.8%) |
| Family history of cancer, n (%) |  |  |  |  |
| No | 49063 (62.5%) | 16223 (62.0%) | 16265 (62.1%) | 16575 (63.4%) |
| Yes | 29445 (37.5%) | 9947 (38.0%) | 9910 (37.9%) | 9588 (36.6%) |
| Total wear days, mean (SD) | 6.72 (0.709) | 6.70 (0.747) | 6.73 (0.692) | 6.74 (0.686) |
| Seasonality, n (%) |  |  |  |  |
| Autumn | 23285 (29.7%) | 7643 (29.2%) | 7902 (30.2%) | 7740 (29.6%) |
| Spring | 17873 (22.8%) | 5987 (22.9%) | 5877 (22.5%) | 6009 (23.0%) |
| Summer | 20730 (26.4%) | 7038 (26.9%) | 6756 (25.8%) | 6936 (26.5%) |
| Winter | 16620 (21.2%) | 5502 (21.0%) | 5640 (21.5%) | 5478 (20.9%) |
| TVPA, mg, mean (SD) | 38.4 (11.9) | 44.8 (12.4) | 37.8 (10.1) | 32.5 (9.45) |
| MVPA, hour/day, mean (SD) | 0.517 (0.447) | 0.589 (0.510) | 0.523 (0.434) | 0.438 (0.374) |
| LPA, hour/day, mean (SD) | 5.27 (1.58) | 6.63 (1.41) | 5.21 (0.995) | 3.96 (0.960) |

CVD, cardiovascular disease; TVPA, total volume of physical activity; MVPA, moderate- to- vigorous- intensity physical activity; LPA, light- intensity physical activity; ST, sedentary time; mg, milligravity; Health diet score was calculated based on self- reported servings of fruits, vegetables, whole grains, vegetable oil, fish, dairy, refined grains, unprocessed meats, processed meats and sugar- sweetened beverages. More details can be found in Additional file 1: Table S4.

Townsend Index (including measures of unemployment, non- car ownership, non- home ownership and household overcrowding), derived from respondents’ postcode was used as an indicator of area- level SES.

Employment status is categorized as employed (includes paid employment or self- employed, paid or voluntary work or student), retired, and inactive (includes looking after home and/or family, unable to work and unemployed).

Education is categorized as college or University degree, secondary school (includes A levels/AS levels or equivalent, O levels/GCSEs or equivalent, CSEs or equivalent), primary school, and professional qualification (NVQ or HND or HNC or equivalent, other professional qualifications).

**Table S11. Joint associations of accelerometer-measured TVPA, MVPA, LPA, and ST with frailty status on all-cause mortality**

| **Frailty index** | **TVPA** | **Case** | **Total number** | **HR (95%CI)** | **P for interaction** |
| --- | --- | --- | --- | --- | --- |
| robust | top tertile | 305 | 18575 | Ref. | <0.001 |
| pre-frailty | top tertile | 130 | 7072 | 0.99 (0.81-1.22) |  |
| frailty | top tertile | 17 | 522 | 1.49 (0.91-2.43) |  |
| robust | medium tertile | 381 | 16895 | 1.09 (0.94-1.27) |  |
| pre-frailty | medium tertile | 287 | 8409 | 1.46 (1.24-1.73) |  |
| frailty | medium tertile | 32 | 865 | 1.43 (0.99-2.07) |  |
| robust | low tertile | 657 | 14266 | 1.71 (1.49-1.97) |  |
| pre-frailty | low tertile | 621 | 10098 | 2.01 (1.74-2.33) |  |
| frailty | low tertile | 188 | 1806 | 3.05 (2.50-3.71) |  |
| **Frailty index** | **MVPA** | **Case** | **Total number** | **HR (95%CI)** | **P for interaction** |
| robust | top tertile | 402 | 18798 | Ref. | <0.001 |
| pre-frailty | top tertile | 207 | 6877 | 1.22 (1.03-1.44) |  |
| frailty | top tertile | 15 | 461 | 1.03 (0.61-1.73) |  |
| robust | medium tertile | 446 | 17041 | 1.15 (1.00-1.32) |  |
| pre-frailty | medium tertile | 289 | 8306 | 1.31 (1.12-1.53) |  |
| frailty | medium tertile | 39 | 793 | 1.59 (1.14-2.21) |  |
| robust | low tertile | 495 | 13897 | 1.39 (1.21-1.59) |  |
| pre-frailty | low tertile | 542 | 10396 | 1.71 (1.48-1.96) |  |
| frailty | low tertile | 183 | 1939 | 2.65 (2.19-3.21) |  |
| **Frailty index** | **LPA** | **Case** | **Total number** | **HR (95%CI)** | **P for interaction** |
| robust | top tertile | 375 | 16987 | Ref. | <0.001 |
| pre-frailty | top tertile | 237 | 8316 | 1.11 (0.94-1.31) |  |
| frailty | top tertile | 44 | 866 | 1.72 (1.26-2.36) |  |
| robust | medium tertile | 428 | 16741 | 1.03 (0.88-1.19) |  |
| pre-frailty | medium tertile | 314 | 8489 | 1.25 (1.07-1.47) |  |
| frailty | medium tertile | 57 | 925 | 1.73 (1.30-2.30) |  |
| robust | low tertile | 540 | 16008 | 1.25 (1.07-1.47) |  |
| pre-frailty | low tertile | 487 | 8774 | 1.60 (1.36-1.88) |  |
| frailty | low tertile | 136 | 1402 | 2.26 (1.81-2.83) |  |
| **Frailty index** | **ST** | **Case** | **Total number** | **HR (95%CI)** | **P for interaction** |
| robust | top tertile | 397 | 16590 | Ref. | <0.001 |
| pre-frailty | top tertile | 259 | 8542 | 1.06 (0.90-1.24) |  |
| frailty | top tertile | 60 | 1038 | 1.66 (1.26-2.19) |  |
| robust | medium tertile | 456 | 16586 | 1.08 (0.94-1.23) |  |
| pre-frailty | medium tertile | 343 | 8574 | 1.27 (1.09-1.47) |  |
| frailty | medium tertile | 72 | 1015 | 1.74 (1.34-2.25) |  |
| robust | low tertile | 490 | 16560 | 1.18 (1.03-1.35) |  |
| pre-frailty | low tertile | 436 | 8463 | 1.54 (1.34-1.78) |  |
| frailty | low tertile | 105 | 1140 | 2.08 (1.66-2.61) |  |

TVPA, total volume of physical activity; MVPA, moderate- to- vigorous- intensity physical activity; LPA, light- intensity physical activity; ST, sedentary time; HR, hazard ratios; CI, confidence interval.

Model adjusted for age at accelerometer measurement, sex, assessment center, body mass index, ethnicity, education, employment, household income, Townsend deprivation index, smoking status, alcohol drinking frequency, sleep duration, healthy diet score, family history of diabetes, family history of CVD, family history of cancer, seasonality, and total wear days. MVPA and LPA models were further adjusted for ST, ST model was further adjusted for MVPA, while TVPA model was not further adjusted.

**Table S12. Joint associations of accelerometer-measured TVPA, MVPA, LPA, and ST with frailty status on all-cause mortality (remove deaths within first 2 years, n=78062)**

| **Frailty index** | **TVPA** | **Case** | **Total number** | **HR (95%CI)** | **P for interaction** |
| --- | --- | --- | --- | --- | --- |
| robust | top tertile | 259 | 18479 | Ref. | <0.001 |
| pre-frailty | top tertile | 104 | 7022 | 0.93 (0.74-1.17) |  |
| frailty | top tertile | 15 | 520 | 1.54 (0.91-2.60) |  |
| robust | medium tertile | 342 | 16827 | 1.15 (0.98-1.35) |  |
| pre-frailty | medium tertile | 241 | 8342 | 1.44 (1.20-1.72) |  |
| frailty | medium tertile | 27 | 851 | 1.43 (0.96-2.14) |  |
| robust | low tertile | 532 | 14220 | 1.62 (1.39-1.89) |  |
| pre-frailty | low tertile | 504 | 10026 | 1.90 (1.62-2.23) |  |
| frailty | low tertile | 148 | 1775 | 2.79 (2.25-3.47) |  |
| **Frailty index** | **MVPA** | **Case** | **Total number** | **HR (95%CI)** | **P for interaction** |
| robust | top tertile | 345 | 18695 | Ref. | <0.001 |
| pre-frailty | top tertile | 173 | 6820 | 1.19 (0.99-1.43) |  |
| frailty | top tertile | 13 | 458 | 1.04 (0.60-1.81) |  |
| robust | medium tertile | 376 | 16928 | 1.13 (0.97-1.31) |  |
| pre-frailty | medium tertile | 237 | 8235 | 1.25 (1.05-1.48) |  |
| frailty | medium tertile | 31 | 783 | 1.47 (1.06-2.13) |  |
| robust | low tertile | 412 | 13903 | 1.34 (1.15-1.55) |  |
| pre-frailty | low tertile | 439 | 10335 | 1.61 (1.38-1.87) |  |
| frailty | low tertile | 146 | 1905 | 2.47 (2.00-3.05) |  |
| **Frailty index** | **LPA** | **Case** | **Total number** | **HR (95%CI)** | **P for interaction** |
| robust | top tertile | 331 | 16901 | Ref. | <0.001 |
| pre-frailty | top tertile | 198 | 8262 | 1.05 (0.88-1.25) |  |
| frailty | top tertile | 37 | 858 | 1.63 (1.16-2.30) |  |
| robust | medium tertile | 363 | 16668 | 0.98 (0.83-1.15) |  |
| pre-frailty | medium tertile | 257 | 8427 | 1.15 (0.97-1.37) |  |
| frailty | medium tertile | 51 | 913 | 1.75 (1.29-2.38) |  |
| robust | low tertile | 439 | 15957 | 1.15 (0.97-1.37) |  |
| pre-frailty | low tertile | 394 | 8701 | 1.46 (1.23-1.75) |  |
| frailty | low tertile | 102 | 1375 | 1.92 (1.49-2.46) |  |
| **Frailty index** | **ST** | **Case** | **Total number** | **HR (95%CI)** | **P for interaction** |
| robust | top tertile | 347 | 16525 | Ref. | <0.001 |
| pre-frailty | top tertile | 206 | 8483 | 0.97 (0.81-1.15) |  |
| frailty | top tertile | 48 | 1025 | 1.54 (1.13-2.09) |  |
| robust | medium tertile | 392 | 16505 | 1.06 (0.92-1.23) |  |
| pre-frailty | medium tertile | 289 | 8514 | 1.23 (1.05-1.44) |  |
| frailty | medium tertile | 58 | 1002 | 1.62 (1.22-2.15) |  |
| robust | low tertile | 394 | 16496 | 1.10 (0.94-1.27) |  |
| pre-frailty | low tertile | 354 | 8393 | 1.46 (1.25-1.70) |  |
| frailty | low tertile | 84 | 1119 | 1.95 (1.52-2.50) |  |

TVPA, total volume of physical activity; MVPA, moderate- to- vigorous- intensity physical activity; LPA, light- intensity physical activity; ST, sedentary time; HR, hazard ratios; CI, confidence interval.

Model adjusted for age at accelerometer measurement, sex, assessment center, body mass index, ethnicity, education, employment, household income, Townsend deprivation index, smoking status, alcohol drinking frequency, sleep duration, healthy diet score, family history of diabetes, family history of CVD, family history of cancer, seasonality, and total wear days. MVPA and LPA models were further adjusted for ST, ST model was further adjusted for MVPA, while TVPA model was not further adjusted.

**Table S13. Joint associations of accelerometer-measured TVPA, MVPA, LPA, and ST with frailty status on all-cause mortality (remove missing values, n=64771)**

| **Frailty index** | **TVPA** | **Case** | **Total number** | **HR (95%CI)** | **P for interaction** |
| --- | --- | --- | --- | --- | --- |
| robust | top tertile | 247 | 15476 | Ref. | <0.001 |
| pre-frailty | top tertile | 112 | 5727 | 1.08 (0.87-1.36) |  |
| frailty | top tertile | 10 | 387 | 1.21 (0.90-2.28) |  |
| robust | medium tertile | 317 | 14134 | 1.11 (0.94-1.32) |  |
| pre-frailty | medium tertile | 215 | 6788 | 1.39 (1.15-1.67) |  |
| frailty | medium tertile | 26 | 668 | 1.53 (1.01-2.30) |  |
| robust | low tertile | 556 | 12044 | 1.77 (1.52-2.07) |  |
| pre-frailty | low tertile | 490 | 8167 | 2.00 (1.70-2.35) |  |
| frailty | low tertile | 144 | 1380 | 3.00 (2.40-3.75) |  |
| **Frailty index** | **MVPA** | **Case** | **Total number** | **HR (95%CI)** | **P for interaction** |
| robust | top tertile | 335 | 15634 | Ref. | <0.001 |
| pre-frailty | top tertile | 172 | 5596 | 1.22 (1.01-1.47) |  |
| frailty | top tertile | 10 | 359 | 0.85 (0.45-1.60) |  |
| robust | medium tertile | 362 | 14214 | 1.11 (0.95-1.29) |  |
| pre-frailty | medium tertile | 238 | 6708 | 1.32 (1.11-1.56) |  |
| frailty | medium tertile | 34 | 589 | 1.83 (1.28-2.61) |  |
| robust | low tertile | 423 | 11806 | 1.39 (1.19-1.61) |  |
| pre-frailty | low tertile | 407 | 8378 | 1.56 (1.33-1.82) |  |
| frailty | low tertile | 136 | 1487 | 2.42 (1.94-3.01) |  |
| **Frailty index** | **LPA** | **Case** | **Total number** | **HR (95%CI)** | **P for interaction** |
| robust | top tertile | 308 | 14160 | Ref. | <0.001 |
| pre-frailty | top tertile | 194 | 6766 | 1.14 (0.95-1.36) |  |
| frailty | top tertile | 31 | 651 | 1.64 (1.13-2.38) |  |
| robust | medium tertile | 360 | 14054 | 1.07 (0.91-1.27) |  |
| pre-frailty | medium tertile | 238 | 6820 | 1.22 (1.02-1.46) |  |
| frailty | medium tertile | 43 | 729 | 1.65 (1.19-2.30) |  |
| robust | low tertile | 452 | 13440 | 1.31 (1.10-1.57) |  |
| pre-frailty | low tertile | 385 | 7096 | 1.62 (1.35-1.94) |  |
| frailty | low tertile | 106 | 1055 | 2.34 (1.82-3.01) |  |
| **Frailty index** | **ST** | **Case** | **Total number** | **HR (95%CI)** | **P for interaction** |
| robust | top tertile | 332 | 13867 | Ref. | <0.001 |
| pre-frailty | top tertile | 213 | 6928 | 1.08 (0.91-1.29) |  |
| frailty | top tertile | 46 | 796 | 1.65 (1.21-2.26) |  |
| robust | medium tertile | 379 | 13883 | 1.08 (0.94-1.26) |  |
| pre-frailty | medium tertile | 262 | 6930 | 1.20 (1.02-1.42) |  |
| frailty | medium tertile | 57 | 777 | 1.76 (1.31-2.34) |  |
| robust | low tertile | 409 | 13904 | 1.19 (1.02-1.38) |  |
| pre-frailty | low tertile | 342 | 6824 | 1.52 (1.30-1.78) |  |
| frailty | low tertile | 77 | 862 | 1.97 (1.52-2.55) |  |

TVPA, total volume of physical activity; MVPA, moderate- to- vigorous- intensity physical activity; LPA, light- intensity physical activity; ST, sedentary time; HR, hazard ratios; CI, confidence interval.

Model adjusted for age at accelerometer measurement, sex, assessment center, body mass index, ethnicity, education, employment, household income, Townsend deprivation index, smoking status, alcohol drinking frequency, sleep duration, healthy diet score, family history of diabetes, family history of CVD, family history of cancer, seasonality, and total wear days. MVPA and LPA models were further adjusted for ST, ST model was further adjusted for MVPA, while TVPA model was not further adjusted.

**Table S14. Joint associations of accelerometer-measured TVPA, MVPA, LPA, and ST with frailty status on all-cause mortality (remove night shift workers, n=75571)**

| **Frailty index** | **TVPA** | **Case** | **Total number** | **HR (95%CI)** | **P for interaction** |
| --- | --- | --- | --- | --- | --- |
| robust | top tertile | 295 | 17879 | Ref. | <0.001 |
| pre-frailty | top tertile | 127 | 6805 | 1.00 (0.81-1.23) |  |
| frailty | top tertile | 17 | 506 | 1.52 (0.93-2.48) |  |
| robust | medium tertile | 373 | 16263 | 1.10 (0.95-1.29) |  |
| pre-frailty | medium tertile | 284 | 8095 | 1.50 (1.27-1.77) |  |
| frailty | medium tertile | 31 | 832 | 1.43 (0.98-2.08) |  |
| robust | low tertile | 640 | 13696 | 1.73 (1.50-2.00) |  |
| pre-frailty | low tertile | 608 | 9742 | 2.03 (1.75-2.35) |  |
| frailty | low tertile | 187 | 1753 | 3.11 (2.55-3.79) |  |
| **Frailty index** | **MVPA** | **Case** | **Total number** | **HR (95%CI)** | **P for interaction** |
| robust | top tertile | 395 | 18111 | Ref. | <0.001 |
| pre-frailty | top tertile | 202 | 6632 | 1.21 (1.02-1.43) |  |
| frailty | top tertile | 15 | 443 | 1.05 (0.63-1.77) |  |
| robust | medium tertile | 433 | 16419 | 1.13 (0.99-1.30) |  |
| pre-frailty | medium tertile | 285 | 7997 | 1.31 (1.12-1.53) |  |
| frailty | medium tertile | 39 | 768 | 1.60 (1.15-2.24) |  |
| robust | low tertile | 480 | 13308 | 1.37 (1.19-1.58) |  |
| pre-frailty | low tertile | 532 | 10013 | 1.69 (1.47-1.94) |  |
| frailty | low tertile | 181 | 1880 | 2.63 (2.17-3.19) |  |
| **Frailty index** | **LPA** | **Case** | **Total number** | **HR (95%CI)** | **P for interaction** |
| robust | top tertile | 363 | 16359 | Ref. | <0.001 |
| pre-frailty | top tertile | 232 | 7999 | 1.12 (0.95-1.33) |  |
| frailty | top tertile | 43 | 832 | 1.73 (1.26-2.38) |  |
| robust | medium tertile | 416 | 16092 | 1.04 (0.90-1.21) |  |
| pre-frailty | medium tertile | 306 | 8186 | 1.27 (1.08-1.50) |  |
| frailty | medium tertile | 57 | 894 | 1.79 (1.34-2.39) |  |
| robust | low tertile | 529 | 15387 | 1.29 (1.09-1.51) |  |
| pre-frailty | low tertile | 481 | 8457 | 1.65 (1.40-1.94) |  |
| frailty | low tertile | 135 | 1365 | 2.34 (1.87-2.93) |  |
| **Frailty index** | **ST** | **Case** | **Total number** | **HR (95%CI)** | **P for interaction** |
| robust | top tertile | 389 | 15966 | Ref. | <0.001 |
| pre-frailty | top tertile | 256 | 8238 | 1.07 (0.91-1.26) |  |
| frailty | top tertile | 61 | 1009 | 1.71 (1.30-2.25) |  |
| robust | medium tertile | 441 | 15941 | 1.08 (0.94-1.23) |  |
| pre-frailty | medium tertile | 336 | 8250 | 1.27 (1.10-1.48) |  |
| frailty | medium tertile | 71 | 984 | 1.76 (1.36-2.29) |  |
| robust | low tertile | 478 | 15931 | 1.19 (1.03-1.36) |  |
| pre-frailty | low tertile | 427 | 8154 | 1.55 (1.35-1.79) |  |
| frailty | low tertile | 103 | 1098 | 2.09 (1.66-2.62) |  |

TVPA, total volume of physical activity; MVPA, moderate- to- vigorous- intensity physical activity; LPA, light- intensity physical activity; ST, sedentary time; HR, hazard ratios; CI, confidence interval.

Model adjusted for age at accelerometer measurement, sex, assessment center, body mass index, ethnicity, education, employment, household income, Townsend deprivation index, smoking status, alcohol drinking frequency, sleep duration, healthy diet score, family history of diabetes, family history of CVD, family history of cancer, seasonality, and total wear days. MVPA and LPA models were further adjusted for ST, ST model was further adjusted for MVPA, while TVPA model was not further adjusted.

**Table S15. Joint associations of accelerometer-measured TVPA, MVPA, LPA, and ST with frailty status on all-cause mortality (awake time: 07:00-21:00)**

| **Frailty index** | **TVPA** | **Case** | **Total number** | **HR (95%CI)** | **P for interaction** |
| --- | --- | --- | --- | --- | --- |
| robust | top tertile | 304 | 18587 | Ref. | <0.001 |
| pre-frailty | top tertile | 132 | 7060 | 1.02 (0.83-1.25) |  |
| frailty | top tertile | 15 | 522 | 1.34 (0.80-2.25) |  |
| robust | medium tertile | 392 | 16841 | 1.15 (0.99-1.34) |  |
| pre-frailty | medium tertile | 288 | 8458 | 1.48 (1.26-1.75) |  |
| frailty | medium tertile | 33 | 870 | 1.48 (1.03-2.12) |  |
| robust | low tertile | 647 | 14308 | 1.75 (1.52-2.02) |  |
| pre-frailty | low tertile | 618 | 10061 | 2.08 (1.80-2.41) |  |
| frailty | low tertile | 189 | 1801 | 3.18 (2.61-3.86) |  |
| **Frailty index** | **MVPA** | **Case** | **Total number** | **HR (95%CI)** | **P for interaction** |
| robust | top tertile | 401 | 18769 | Ref. | <0.001 |
| pre-frailty | top tertile | 211 | 6876 | 1.24 (1.05-1.46) |  |
| frailty | top tertile | 15 | 466 | 1.00 (0.59-1.68) |  |
| robust | medium tertile | 449 | 17048 | 1.16 (1.01-1.33) |  |
| pre-frailty | medium tertile | 287 | 8312 | 1.30 (1.11-1.52) |  |
| frailty | medium tertile | 39 | 793 | 1.61 (1.15-2.24) |  |
| robust | low tertile | 493 | 13919 | 1.39 (1.21-1.60) |  |
| pre-frailty | low tertile | 540 | 10391 | 1.71 (1.49-1.96) |  |
| frailty | low tertile | 183 | 1934 | 2.67 (2.20-3.23) |  |
| **Frailty index** | **LPA** | **Case** | **Total number** | **HR (95%CI)** | **P for interaction** |
| robust | top tertile | 389 | 16925 | Ref. | <0.001 |
| pre-frailty | top tertile | 240 | 8360 | 1.08 (0.92-1.27) |  |
| frailty | top tertile | 46 | 874 | 1.74 (1.27-2.36) |  |
| robust | medium tertile | 422 | 16719 | 1.02 (0.88-1.19) |  |
| pre-frailty | medium tertile | 309 | 8506 | 1.23 (1.05-1.45) |  |
| frailty | medium tertile | 56 | 929 | 1.68 (1.26-2.25) |  |
| robust | low tertile | 532 | 16092 | 1.28 (1.09-1.51) |  |
| pre-frailty | low tertile | 489 | 8713 | 1.67 (1.42-1.97) |  |
| frailty | low tertile | 135 | 1390 | 2.33 (1.86-2.92) |  |
| **Frailty index** | **ST** | **Case** | **Total number** | **HR (95%CI)** | **P for interaction** |
| robust | top tertile | 411 | 16551 | Ref. | <0.001 |
| pre-frailty | top tertile | 270 | 8576 | 1.07 (0.91-1.25) |  |
| frailty | top tertile | 59 | 1047 | 1.55 (1.17-2.04) |  |
| robust | medium tertile | 440 | 16568 | 1.00 (0.88-1.15) |  |
| pre-frailty | medium tertile | 333 | 8579 | 1.18 (1.02-1.37) |  |
| frailty | medium tertile | 75 | 1026 | 1.78 (1.38-2.29) |  |
| robust | low tertile | 492 | 16617 | 1.16 (1.01-1.33) |  |
| pre-frailty | low tertile | 435 | 8424 | 1.51 (1.31-1.74) |  |
| frailty | low tertile | 103 | 1120 | 2.00 (1.59-2.50) |  |

TVPA, total volume of physical activity; MVPA, moderate- to- vigorous- intensity physical activity; LPA, light- intensity physical activity; ST, sedentary time; HR, hazard ratios; CI, confidence interval.

Model adjusted for age at accelerometer measurement, sex, assessment center, body mass index, ethnicity, education, employment, household income, Townsend deprivation index, smoking status, alcohol drinking frequency, sleep duration, healthy diet score, family history of diabetes, family history of CVD, family history of cancer, seasonality, and total wear days. MVPA and LPA models were further adjusted for ST, ST model was further adjusted for MVPA, while TVPA model was not further adjusted.

**Table S16. Joint associations of accelerometer-measured TVPA, MVPA, LPA, and ST with frailty status on all-cause mortality (awake time: 08:00-20:00)**

| **Frailty index** | **TVPA** | **Case** | **Total number** | **HR (95%CI)** | **P for interaction** |
| --- | --- | --- | --- | --- | --- |
| robust | top tertile | 316 | 18466 | Ref. | <0.001 |
| pre-frailty | top tertile | 138 | 7160 | 1.00 (0.82-1.23) |  |
| frailty | top tertile | 15 | 543 | 1.24 (0.74-2.08) |  |
| robust | medium tertile | 398 | 16848 | 1.15 (0.99-1.33) |  |
| pre-frailty | medium tertile | 290 | 8428 | 1.47 (1.25-1.73) |  |
| frailty | medium tertile | 39 | 893 | 1.65 (1.18-2.32) |  |
| robust | low tertile | 629 | 14422 | 1.72 (1.50-1.97) |  |
| pre-frailty | low tertile | 610 | 9991 | 2.06 (1.79-2.38) |  |
| frailty | low tertile | 183 | 1757 | 3.11 (2.55-3.78) |  |
| **Frailty index** | **MVPA** | **Case** | **Total number** | **HR (95%CI)** | **P for interaction** |
| robust | top tertile | 410 | 18735 | Ref. | <0.001 |
| pre-frailty | top tertile | 219 | 6918 | 1.25 (1.06-1.47) |  |
| frailty | top tertile | 19 | 468 | 1.24 (0.78-1.97) |  |
| robust | medium tertile | 443 | 17053 | 1.12 (0.98-1.29) |  |
| pre-frailty | medium tertile | 287 | 8353 | 1.27 (1.09-1.49) |  |
| frailty | medium tertile | 37 | 811 | 1.48 (1.05-2.08) |  |
| robust | low tertile | 490 | 13948 | 1.38 (1.20-1.58) |  |
| pre-frailty | low tertile | 532 | 10308 | 1.67 (1.45-1.92) |  |
| frailty | low tertile | 181 | 1914 | 2.61 (2.15-3.16) |  |
| **Frailty index** | **LPA** | **Case** | **Total number** | **HR (95%CI)** | **P for interaction** |
| robust | top tertile | 389 | 16811 | Ref. | <0.001 |
| pre-frailty | top tertile | 234 | 8470 | 1.04 (0.88-1.23) |  |
| frailty | top tertile | 45 | 887 | 1.69 (1.24-2.31) |  |
| robust | medium tertile | 435 | 16626 | 1.09 (0.94-1.28) |  |
| pre-frailty | medium tertile | 339 | 8569 | 1.39 (1.18-1.63) |  |
| frailty | medium tertile | 58 | 975 | 1.73 (1.30-2.31) |  |
| robust | low tertile | 519 | 16299 | 1.38 (1.16-1.64) |  |
| pre-frailty | low tertile | 465 | 8540 | 1.78 (1.50-2.12) |  |
| frailty | low tertile | 134 | 1331 | 2.59 (2.06-3.27) |  |
| **Frailty index** | **ST** | **Case** | **Total number** | **HR (95%CI)** | **P for interaction** |
| robust | top tertile | 409 | 16619 | Ref. | <0.001 |
| pre-frailty | top tertile | 268 | 8529 | 1.09 (0.93-1.27) |  |
| frailty | top tertile | 59 | 1038 | 1.63 (1.24-2.16) |  |
| robust | medium tertile | 455 | 16458 | 1.06 (0.93-1.21) |  |
| pre-frailty | medium tertile | 339 | 8664 | 1.20 (1.04-1.39) |  |
| frailty | medium tertile | 74 | 1043 | 1.71 (1.33-2.20) |  |
| robust | low tertile | 479 | 16659 | 1.16 (1.02-1.33) |  |
| pre-frailty | low tertile | 431 | 8386 | 1.55 (1.34-1.78) |  |
| frailty | low tertile | 104 | 1112 | 2.09 (1.67-2.62) |  |

TVPA, total volume of physical activity; MVPA, moderate- to- vigorous- intensity physical activity; LPA, light- intensity physical activity; ST, sedentary time; HR, hazard ratios; CI, confidence interval.

Model adjusted for age at accelerometer measurement, sex, assessment center, body mass index, ethnicity, education, employment, household income, Townsend deprivation index, smoking status, alcohol drinking frequency, sleep duration, healthy diet score, family history of diabetes, family history of CVD, family history of cancer, seasonality, and total wear days. MVPA and LPA models were further adjusted for ST, ST model was further adjusted for MVPA, while TVPA model was not further adjusted.

**Table S17. Joint associations of accelerometer-measured MVPA and LPA with frailty status on all-cause mortality (further mutually adjusted for MVPA or LPA)**

| **Frailty index** | **MVPA** | **Case** | **Total number** | **HR (95%CI)** | **HR (95%CI) *** | **P for interaction** |
| --- | --- | --- | --- | --- | --- | --- |
| robust | top tertile | 402 | 18798 | Ref. | Ref. | <0.001 |
| pre-frailty | top tertile | 207 | 6877 | 1.22 (1.03-1.44) | 1.21 (1.02-1.43) |  |
| frailty | top tertile | 15 | 461 | 1.03 (0.61-1.73) | 1.02 (0.61-1.71) |  |
| robust | medium tertile | 446 | 17041 | 1.15 (1.00-1.32) | 1.17 (1.02-1.34) |  |
| pre-frailty | medium tertile | 289 | 8306 | 1.31 (1.12-1.53) | 1.33 (1.14-1.55) |  |
| frailty | medium tertile | 39 | 793 | 1.59 (1.14-2.21) | 1.59 (1.14-2.23) |  |
| robust | low tertile | 495 | 13897 | 1.39 (1.21-1.59) | 1.41 (1.23-1.62) |  |
| pre-frailty | low tertile | 542 | 10396 | 1.71 (1.48-1.96) | 1.71 (1.49-1.97) |  |
| frailty | low tertile | 183 | 1939 | 2.65 (2.19-3.21) | 2.62 (2.16-3.18) |  |
| **Frailty index** | **LPA** | **Case** | **Total number** | **HR (95%CI)** | **HR (95%CI) *** | **P for interaction** |
| robust | top tertile | 375 | 16987 | Ref. | Ref. | <0.001 |
| pre-frailty | top tertile | 237 | 8316 | 1.11 (0.94-1.31) | 1.09 (0.92-1.28) |  |
| frailty | top tertile | 44 | 866 | 1.72 (1.26-2.36) | 1.64 (1.20-2.25) |  |
| robust | medium tertile | 428 | 16741 | 1.03 (0.88-1.19) | 1.06 (0.91-1.23) |  |
| pre-frailty | medium tertile | 314 | 8489 | 1.25 (1.07-1.47) | 1.26 (1.08-1.49) |  |
| frailty | medium tertile | 57 | 925 | 1.73 (1.30-2.30) | 1.69 (1.27-2.25) |  |
| robust | low tertile | 540 | 16008 | 1.25 (1.07-1.47) | 1.29 (1.10-1.52) |  |
| pre-frailty | low tertile | 487 | 8774 | 1.60 (1.36-1.88) | 1.60 (1.36-1.88) |  |
| frailty | low tertile | 136 | 1402 | 2.26 (1.81-2.83) | 2.18 (1.75-2.73) |  |

MVPA, moderate- to- vigorous- intensity physical activity; LPA, light- intensity physical activity; ST, sedentary time; HR, hazard ratios; CI, confidence interval.

Model adjusted for age at accelerometer measurement, sex, assessment center, body mass index, ethnicity, education, employment, household income, Townsend deprivation index, smoking status, alcohol drinking frequency, sleep duration, healthy diet score, family history of diabetes, family history of CVD, family history of cancer, seasonality, total wear days, and ST. *Model further mutually adjusted for MVPA or LPA.

**Table S18. Analyses on interaction of accelerometer-measured physical activity and sedentary and pre-frailty with all-cause mortality**

|  | **Robust** | **Pre-frailty** | **Multiplicative interaction** | **Additive interaction** | | |
| --- | --- | --- | --- | --- | --- | --- |
|  |  |  |  | **RERI** | **AP** | **S** |
|  | HR (95%CI) | HR (95%CI) | HR (95%CI) |  |  |  |
| **TVPA** |  | | | | | |
| Top tertile + medium tertile | Ref. | 1.21 (1.07, 1.37) | 0.97 (0.82, 1.14) | 0.07 (-0.17, 0.30) | 0.03 (-0.09, 0.16) | 1.08 (0.81, 1.43) |
| Low tertile | 1.62 (1.45, 1.81) | 1.90 (1.69, 2.14) |  |  |  |  |
| **MVPA** |  | | | | | |
| Top tertile + medium tertile | Ref. | 1.18 (1.05, 1.32) | 1.04 (0.88, 1.23) | 0.11 (-0.10, 0.32) | 0.07 (-0.06, 0.20) | 1.24 (0.80, 1.92) |
| Low tertile | 1.29 (1.15, 1.44) | 1.57 (1.40, 1.77) |  |  |  |  |
| **LPA** |  | | | | | |
| Top tertile + medium tertile | Ref. | 1.17 (1.05, 1.30) | 1.09 (0.93, 1.29) | 0.17 (-0.04, 0.38) | 0.11 (-0.02, 0.24) | 1.44 (0.87, 2.38) |
| Low tertile | 1.22 (1.07, 1.38) | 1.56 (1.37, 1.77) |  |  |  |  |
| **ST** |  | | | | | |
| Low tertile + medium tertile | Ref. | 1.12 (1.01, 1.25) | 1.17 (0.99, 1.38) | 0.23 (0.03, 0.43) | 0.15 (0.03, 0.28) | 1.90 (0.93, 3.89) |
| Top tertile | 1.13 (1.01, 1.27) | 1.48 (1.31, 1.67) |  |  |  |  |

TVPA, total volume of physical activity; MVPA, moderate- to- vigorous- intensity physical activity; LPA, light- intensity physical activity; ST, sedentary time; RERI, relative excess risk due to interaction; AP, attributable proportion due to interaction; S, the synergy index; HR, hazard ratio; CI, confidence interval.

Model adjusted for age at accelerometer measurement, sex, assessment center, body mass index, ethnicity, education, employment, household income, Townsend deprivation index, smoking status, alcohol drinking frequency, sleep duration, healthy diet score, family history of diabetes, family history of CVD, family history of cancer, seasonality, and total wear days. MVPA and LPA models were further adjusted for ST, ST model was further adjusted for MVPA, while TVPA model was not further adjusted.

**Table S19. Analyses on interaction of accelerometer-measured physical activity and sedentary and frailty with all-cause mortality**

|  | **Robust** | **Frailty** | **Multiplicative interaction** | **Additive interaction** | | |
| --- | --- | --- | --- | --- | --- | --- |
|  |  |  |  | **RERI** | **AP** | **S** |
|  | HR (95%CI) | HR (95%CI) | HR (95%CI) |  |  |  |
| **TVPA** |  | | | | | |
| Top tertile + medium tertile | Ref. | 1.44 (1.07, 1.94) | 1.30 (0.93, 1.82) | 0.99 (0.36, 1.62) | 0.32 (0.15, 0.50) | 1.92 (1.21, 3.04) |
| Low tertile | 1.64 (1.46, 1.83) | 3.07 (2.55, 3.69) |  |  |  |  |
| **MVPA** |  | | | | | |
| Top tertile + medium tertile | Ref. | 1.35 (1.02, 1.79) | 1.49 (1.08, 2.06) | 0.96 (0.41, 1.51) | 0.37 (0.19, 0.54) | 2.48 (1.29, 4.76) |
| Low tertile | 1.30 (1.16, 1.46) | 2.61 (2.17, 3.14) |  |  |  |  |
| **LPA** |  | | | | | |
| Top tertile + medium tertile | Ref. | 1.77 (1.43, 2.20) | 1.07 (0.81, 1.42) | 0.38 (-0.18, 0.94) | 0.16 (-0.05, 0.37) | 1.36 (0.86, 2.16) |
| Low tertile | 1.28 (1.12, 1.46) | 2.42 (1.96, 3.00) |  |  |  |  |
| **ST** |  | | | | | |
| Low tertile + medium tertile | Ref. | 1.70 (1.40, 2.07) | 1.09 (0.82, 1.44) | 0.27 (-0.24, 0.78) | 0.13 (-0.10, 0.35) | 1.32 (0.78, 2.24) |
| Top tertile | 1.14 (1.01, 1.28) | 2.11 (1.69, 2.63) |  |  |  |  |

TVPA, total volume of physical activity; MVPA, moderate- to- vigorous- intensity physical activity; LPA, light- intensity physical activity; ST, sedentary time; RERI, relative excess risk due to interaction; AP, attributable proportion due to interaction; S, the synergy index; HR, hazard ratio; CI, confidence interval.

Model adjusted for age at accelerometer measurement, sex, assessment center, body mass index, ethnicity, education, employment, household income, Townsend deprivation index, smoking status, alcohol drinking frequency, sleep duration, healthy diet score, family history of diabetes, family history of CVD, family history of cancer, seasonality, and total wear days. MVPA and LPA models were further adjusted for ST, ST model was further adjusted for MVPA, while TVPA model was not further adjusted.

**Table S20. Association between frailty index and all-cause mortality**

|  |  |  | **Model 1** | **Model 2** | **Model 3** |
| --- | --- | --- | --- | --- | --- |
|  | **Deaths** | **N** | **HR (95% CI)** | **HR (95%CI)** | **HR (95%CI)** |
| **Frailty index** |  |  |  |  |  |
| Robust | 1343 | 49736 | Ref. | Ref. | Ref. |
| Pre-frailty | 1038 | 25579 | 1.52 (1.40, 1.64) | 1.23 (1.13, 1.33) | 1.18 (1.09, 1.29) |
| Frailty | 237 | 3193 | 2.83 (2.46, 3.25) | 1.80 (1.55, 2.09) | 1.66 (1.43, 1.93) |
| HR per 1 SD |  |  | 1.34 (1.30, 1.39) | 1.17 (1.13, 1.22) | 1.14 (1.10, 1.19) |
| P for trend |  |  | <0.001 | <0.001 | <0.001 |
| AIC |  |  | 57950.8 | 56086.6 | 55951.3 |
| BIC |  |  | 57962.5 | 56274.4 | 56174.4 |

Model 1: unadjusted model.

Model 2: adjusted for age at accelerometer measurement, sex, assessment center, body mass index, ethnicity, education, employment, household income, Townsend deprivation index, smoking status, alcohol drinking frequency, sleep duration, healthy diet score, family history of diabetes, family history of CVD, family history of cancer.

Model 3: further adjusted for seasonality, total wear days, and TVPA.

TVPA, total volume of physical activity; HR, hazard ratios; CI, confidence interval; SD: standard deviation; AIC, Akaike information criterion; BIC, Bayesian information criterion.

**Table S21. Association between accelerometer-measured TVPA, MVPA, LPA, and ST and all-cause mortality**

|  |  |  | **Model 1** | **Model 2** | **Model 3** |
| --- | --- | --- | --- | --- | --- |
|  | **Deaths** | **N** | **HR (95% CI)** | **HR (95%CI)** | **HR (95%CI)** |
| **TVPA** |  |  |  |  |  |
| Low tertile | 1466 | 26170 | Ref. | Ref. | Ref. |
| Medium tertile | 700 | 26169 | 0.47 (0.43, 0.51) | 0.64 (0.59, 0.71) | 0.66 (0.60, 0.72) |
| Top tertile | 452 | 26169 | 0.30 (0.27, 0.33) | 0.53 (0.48, 0.60) | 0.55 (0.49, 0.61) |
| HR per 1 SD |  |  | 0.49 (0.47, 0.52) | 0.67 (0.63, 0.70) | 0.68 (0.64, 0.71) |
| P for trend |  |  | <0.001 | <0.001 | <0.001 |
| AIC |  |  | 57530.5 | 55992.9 | 55951.3 |
| BIC |  |  | 57542.3 | 56204.2 | 56174.4 |
| **MVPA** |  |  |  |  |  |
| Low tertile | 1220 | 26232 | Ref. | Ref. | Ref. |
| Medium tertile | 774 | 26140 | 0.63 (0.57, 0.69) | 0.76 (0.69, 0.83) | 0.78 (0.71, 0.86) |
| Top tertile | 624 | 26136 | 0.50 (0.46, 0.56) | 0.67 (0.60, 0.74) | 0.69 (0.62, 0.77) |
| HR per 1 SD |  |  | 0.71 (0.68, 0.74) | 0.81 (0.77, 0.86) | 0.83 (0.79, 0.87) |
| P for trend |  |  | <0.001 | <0.001 | <0.001 |
| AIC |  |  | 57958.5 | 56050.3 | 56006.7 |
| BIC |  |  | 57970.3 | 56273.3 | 56241.5 |
| **LPA** |  |  |  |  |  |
| Low tertile | 1163 | 26184 | Ref. | Ref. | Ref. |
| Medium tertile | 799 | 26155 | 0.68 (0.63, 0.75) | 0.79 (0.71, 0.87) | 0.80 (0.72, 0.88) |
| Top tertile | 656 | 26169 | 0.56 (0.51, 0.61) | 0.74 (0.65, 0.84) | 0.75 (0.66, 0.86) |
| HR per 1 SD |  |  | 0.73 (0.70, 0.76) | 0.79 (0.75, 0.84) | 0.81 (0.76, 0.85) |
| P for trend |  |  | <0.001 | <0.001 | <0.001 |
| AIC |  |  | 58022.8 | 56087.3 | 56035.3 |
| BIC |  |  | 58034.6 | 56310.3 | 56270.1 |
| **ST** |  |  |  |  |  |
| Low tertile | 716 | 26170 | Ref. | Ref. | Ref. |
| Medium tertile | 871 | 26175 | 1.22 (1.10, 1.34) | 1.12 (1.01, 1.24) | 1.12 (1.01, 1.23) |
| Top tertile | 1031 | 26163 | 1.45 (1.32, 1.59) | 1.29 (1.17, 1.43) | 1.29 (1.17, 1.42) |
| HR per 1 SD |  |  | 1.20 (1.15, 1.26) | 1.14 (1.08, 1.20) | 1.14 (1.08, 1.19) |
| P for trend |  |  | <0.001 | <0.001 | <0.001 |
| AIC |  |  | 58122.3 | 56050.3 | 56006.7 |
| BIC |  |  | 58134.1 | 56273.3 | 56241.5 |

Model 1: unadjusted model.

Model 2: adjusted for age at accelerometer measurement, sex, assessment center, body mass index, ethnicity, education, employment, household income, Townsend deprivation index, smoking status, alcohol drinking frequency, sleep duration, healthy diet score, family history of diabetes, family history of CVD, family history of cancer, seasonality, and total wear days. MVPA and LPA models were further adjusted for ST, ST model was further adjusted for MVPA, while TVPA model was not further adjusted.

Model 3: further adjusted for frailty index.

TVPA, total volume of physical activity; MVPA, moderate- to- vigorous- intensity physical activity; LPA, light- intensity physical activity; ST, sedentary time; HR, hazard ratios; CI, confidence interval; SD: standard deviation; AIC, Akaike information criterion; BIC, Bayesian information criterion.

**Table S22. Association between various covariates and frailty index using logistic regression models (reference group: robust)**

|  | **Pre-frailty** | **Frailty** |
| --- | --- | --- |
|  | OR (95% CI) | OR (95% CI) |
| Age at accelerometer measurement | 1.02 (1.02-1.02) | 1.03 (1.03-1.04) |
| Sex |  |  |
| female | Ref. | Ref. |
| male | 0.84 (0.82-0.87) | 0.76 (0.70-0.81) |
| Body mass index |  |  |
| <25.0 | Ref. | Ref. |
| 25.0-29.9 | 1.34 (1.30-1.39) | 2.05 (1.85-2.27) |
| >=30.0 | 2.31 (2.22-2.41) | 7.53 (6.82-8.31) |
| Ethnicity |  |  |
| white | Ref. | Ref. |
| other | 1.04 (0.95-1.14) | 1.27 (1.04-1.55) |
| Education |  |  |
| college or university degree | Ref. | Ref. |
| secondary school | 1.28 (1.24-1.32) | 1.81 (1.66-1.98) |
| primary school | 1.98 (1.87-2.10) | 4.42 (3.95-4.95) |
| professional qualification | 1.40 (1.33-1.47) | 2.31 (2.06-2.60) |
| Employment |  |  |
| employed | Ref. | Ref. |
| retired | 1.31 (1.27-1.35) | 1.79 (1.66-1.94) |
| inactive | 1.77 (1.65-1.89) | 7.25 (6.49-8.09) |
| Household income |  |  |
| less than 18000 | Ref. | Ref. |
| 18000 to 30999 | 0.70 (0.67-0.74) | 0.41 (0.37-0.44) |
| 31000 to 51999 | 0.56 (0.53-0.59) | 0.24 (0.21-0.26) |
| 52000 to 100000 | 0.46 (0.44-0.49) | 0.16 (0.14-0.18) |
| greater than 100000 | 0.36 (0.33-0.39) | 0.09 (0.07-0.11) |
| Townsend deprivation index |  |  |
| first quartile | Ref. | Ref. |
| second quartile | 1.04 (0.99-1.08) | 1.17 (1.05-1.32) |
| third quartile | 1.10 (1.05-1.15) | 1.38 (1.23-1.54) |
| fourth quartile | 1.26 (1.21-1.32) | 2.36 (2.13-2.61) |
| Smoking status |  |  |
| never | Ref. | Ref. |
| previous | 1.39 (1.35-1.44) | 1.74 (1.61-1.87) |
| current | 1.57 (1.48-1.68) | 2.73 (2.41-3.09) |
| Alcohol drinking frequency |  |  |
| >= 3 times/week | Ref. | Ref. |
| < 3 times/week | 1.19 (1.16-1.23) | 1.94 (1.79-2.10) |
| never | 1.63 (1.53-1.74) | 4.06 (3.59-4.59) |
| Sleep duration |  |  |
| 7-8 h/day | Ref. | Ref. |
| < 7h/day | 1.64 (1.58-1.70) | 3.12 (2.88-3.37) |
| > 8h/day | 1.63 (1.53-1.73) | 3.60 (3.21-4.03) |
| Healthy diet score |  |  |
| 0-2 | Ref. | Ref. |
| 3-5 | 0.89 (0.86-0.92) | 0.80 (0.74-0.86) |
| >=6 | 0.81 (0.75-0.88) | 0.73 (0.61-0.89) |
| Family history of diabetes |  |  |
| No | Ref. | Ref. |
| Yes | 1.23 (1.19-1.28) | 1.68 (1.55-1.82) |
| Family history of CVD |  |  |
| No | Ref. | Ref. |
| Yes | 1.36 (1.31-1.41) | 2.00 (1.81-2.22) |
| Family history of cancer |  |  |
| No | Ref. | Ref. |
| Yes | 1.09 (1.06-1.13) | 1.11 (1.03-1.19) |

CVD, cardiovascular disease.

Health diet score was calculated based on self- reported servings of fruits, vegetables, whole grains, vegetable oil, fish, dairy, refined grains, unprocessed meats, processed meats and sugar- sweetened beverages. More details can be found in Additional file 1: Table S4.

Townsend Index (including measures of unemployment, non- car ownership, non- home ownership and household overcrowding), derived from respondents’ postcode was used as an indicator of area- level SES.

Employment status is categorized as employed (includes paid employment or self- employed, paid or voluntary work or student), retired, and inactive (includes looking after home and/or family, unable to work and unemployed).

Education is categorized as college or University degree, secondary school (includes A levels/AS levels or equivalent, O levels/GCSEs or equivalent, CSEs or equivalent), primary school, and professional qualification (NVQ or HND or HNC or equivalent, other professional qualifications).

**Table S23. Association between various covariates and all-cause mortality using cox regression models**

|  | **HR (95% CI)** |
| --- | --- |
| Age at accelerometer measurement | 1.12 (1.11-1.12) |
| Sex |  |
| female | Ref. |
| male | 1.94 (1.79-2.09) |
| Body mass index |  |
| <25.0 | Ref. |
| 25.0-29.9 | 1.36 (1.24-1.49) |
| >=30.0 | 1.99 (1.80-2.20) |
| Ethnicity |  |
| white | Ref. |
| other | 0.66 (0.50-0.88) |
| Education |  |
| college or University degree | Ref. |
| secondary school | 1.05 (0.96-1.15) |
| primary school | 2.28 (2.03-2.56) |
| professional qualification | 1.64 (1.46-1.85) |
| Employment |  |
| employed | Ref. |
| retired | 2.41 (2.22-2.62) |
| inactive | 1.84 (1.55-2.19) |
| Household income |  |
| less than 18000 | Ref. |
| 18000 to 30999 | 0.71 (0.64-0.79) |
| 31000 to 51999 | 0.51 (0.46-0.57) |
| 52000 to 100000 | 0.35 (0.31-0.40) |
| greater than 100000 | 0.32 (0.27-0.39) |
| Townsend deprivation index |  |
| first quartile | Ref. |
| second quartile | 1.12 (1.00-1.25) |
| third quartile | 1.10 (0.99-1.23) |
| fourth quartile | 1.21 (1.09-1.35) |
| Smoking status |  |
| never | Ref. |
| previous | 1.70 (1.56-1.84) |
| current | 2.47 (2.17-2.81) |
| Alcohol drinking frequency |  |
| >= 3 times/week | Ref. |
| < 3 times/week | 0.89 (0.82-0.97) |
| never | 1.32 (1.15-1.53) |
| Sleep duration |  |
| 7-8 h/day | Ref. |
| < 7h/day | 1.13 (1.03-1.24) |
| > 8h/day | 1.51 (1.32-1.73) |
| Healthy diet score |  |
| 0-2 | Ref. |
| 3-5 | 0.90 (0.83-0.98) |
| >=6 | 1.10 (0.92-1.31) |
| Family history of diabetes |  |
| No | Ref. |
| Yes | 0.94 (0.86-1.03) |
| Family history of CVD |  |
| No | Ref. |
| Yes | 0.89 (0.81-0.97) |
| Family history of cancer |  |
| No | Ref. |
| Yes | 1.01 (0.94-1.10) |

CVD, cardiovascular disease.

Health diet score was calculated based on self- reported servings of fruits, vegetables, whole grains, vegetable oil, fish, dairy, refined grains, unprocessed meats, processed meats and sugar- sweetened beverages. More details can be found in Additional file 1: Table S4.

Townsend Index (including measures of unemployment, non- car ownership, non- home ownership and household overcrowding), derived from respondents’ postcode was used as an indicator of area- level SES.

Employment status is categorized as employed (includes paid employment or self- employed, paid or voluntary work or student), retired, and inactive (includes looking after home and/or family, unable to work and unemployed).

Education is categorized as college or University degree, secondary school (includes A levels/AS levels or equivalent, O levels/GCSEs or equivalent, CSEs or equivalent), primary school, and professional qualification (NVQ or HND or HNC or equivalent, other professional qualifications).

**Supplementary Method. Estimating the differences in life expectancy**

We combined information from three sources within the same population to estimate lower survival time associated with different levels of frailty index, accelerometer-measured PA/ST, and their interactions.

The reference level was set as “robust” for frailty index, the top tertile for PA or the low tertile for ST.

(1) Sex- and age- specific population mortality rate from the Office for National Statistics [1];

(2) Sex-specific HRs of all-cause mortality in each exposure group versus the reference in UK biobank;

(2) Sex-specific prevalence of each frequency of different levels in UK biobank.

The sex-specific lifetables for each of the 2 exposure groups in frailty index and accelerometer-measured PA/ST, and 8 exposure groups in their combinations were built on the above-mentioned three estimates. Population all-cause mortality rates per 100,000 per sex and per single-year age group were obtained from the Office for National Statistics. We used sex-specific Cox regression models to calculate adjusted hazard ratios for all-cause mortality by exposure groups. Several potential confounders were adjusted in these models, including age at accelerometer measurement, sex, assessment center, body mass index, ethnicity, education, employment, household income, Townsend deprivation index, smoking status, alcohol drinking frequency, sleep duration, healthy diet score, family history of diabetes, family history of CVD, family history of cancer, seasonality, and total wear days. MVPA and LPA models were further adjusted for ST, ST model was further adjusted for MVPA, while TVPA model was not further adjusted. Then we applied the sex- specific HRs to estimate the life expectancy at different age of women and men, separately.

We built the life table starting at age 50 years and ending at 100 years by single-year age intervals. Survival probability was set of 1 at age 50 years and probability of survival between ages x and x + 1 was calculated based on probability of dying (mortality rate) between ages x and x+1 assuming that survivor function declines linearly between ages x and x + 1 [2,3]. The life expectancy at any given age was derived by dividing the total person-years that would be lived beyond age x by the number of persons who survived to that age interval [2].

We inferred the age-specific mortality rates appropriate for our reference group 𝐼𝑅_𝑎0_ as [4]:

$$\boldsymbol{IR}_{\boldsymbol{a}\boldsymbol{0}}\boldsymbol{=}\frac{\boldsymbol{IR}_{\boldsymbol{a}}}{\boldsymbol{(}\boldsymbol{P}_{\boldsymbol{a}\boldsymbol{0}}\boldsymbol{+}\sum_{\boldsymbol{j=1}}^{\boldsymbol{n}} \boldsymbol{P}_{\boldsymbol{aj}}\boldsymbol{\times}\boldsymbol{HR}_{\boldsymbol{aj}}\boldsymbol{)}}$$

(n=2, for 2 exposure groups; n=8, for 8 exposure groups)

Where 𝐼𝑅𝑎 is the population mortality rate for age group 𝑎, 𝑝𝑎𝑗 is the prevalence of exposure group 𝑗, and *H*𝑅𝑎𝑗 is the hazard ratio in comparison of exposure group 𝑗 versus reference group (𝑗 = 0). The age-specific mortality rates in each of the non-reference exposure groups were then inferred in turn by multiplying the age-specific mortality rate for the reference group 𝐼𝑅𝑎0 by the hazard ratios *H*𝑅𝑎𝑗.

Finally, life table was built for each exposure group and the reference group.

**References**

1. Office for National Statistics. Single-year life tables, UK:1980-2020.

<https://www.ons.gov.uk/peoplepopulationandcommunity/birthsdeathsandmarriages/lifeexpectancies/datasets/singleyearlifetablesuk1980to2018/singleyearlifetablesuk>. (Accessed 12 Mar 2024).

2. Arias E. United States life tables, 2008. Natl Vital Stat Rep. 2012;61(3):1-63.

3. Chiang CL, World Health Organization. Life table and mortality analysis. 1979. Publisher: Geneva: World Health Organization.

4. Woloshin S, Schwartz LM, Welch HG. The risk of death by age, sex, and smoking status in the United States: putting health risks in context. J Natl Cancer Inst 2008;100(12):845-53.
